# An *ab initio* Study of the Structure and Energetics of Hydrogen Bonding in Ionic Liquids

**DOI:** 10.3389/fchem.2019.00208

**Published:** 2019-04-10

**Authors:** Kaycee Low, Samuel Y. S. Tan, Ekaterina I. Izgorodina

**Affiliations:** Monash Computational Chemistry Group, School of Chemistry, Monash University, Melbourne, VIC, Australia

**Keywords:** ionic liquids, hydrogen bonding, *ab initio*, protic ionic liquids, interaction energy, electrostatics, dispersion

## Abstract

Unlike typical hydrogen-bonded networks such as water, hydrogen bonded ionic liquids display some unusual characteristics due to the complex interplay of electrostatics, polarization, and dispersion forces in the bulk. Protic ionic liquids in particular contain close-to traditional linear hydrogen bonds that define their physicochemical properties. This work investigates whether hydrogen bonded ionic liquids (HBILs) can be differentiated from aprotic ionic liquids with no linear hydrogen bonds using state-of-the-art *ab initio* calculations. This is achieved through geometry optimizations of a series of single ion pairs of HBILs in the gas phase and an implicit solvent. Using benchmark CCSD(T)/CBS calculations, the electrostatic and dispersion components of the interaction energy of these systems are compared with those of aprotic ionic liquids. The inclusion of the implicit solvent significantly influenced geometries of single ion pairs, with the gas phase shortening the hydrogen bond to reduce electrostatic interactions. HBILs were found to have stronger interactions by at least 10EtMeNH0 kJ mol^−1^ over aprotic ILs, clearly highlighting the electrostatic nature of hydrogen bonding. Geometric and energetic parameters were found to complement each other in determining the extent of hydrogen bonding present in these ionic liquids.

## 1. Introduction

Hydrogen bonding, an intermolecular interaction which is crucial to a myriad of chemical systems, is as diverse and varied as it is common. Although easily identifiable in many systems, the modern definition of what constitutes hydrogen bonding is not straightforward. The IUPAC recommend six requirements for hydrogen bonding, which cumulate in the definition, “*an attractive interaction between a hydrogen atom from a molecule or molecular fragment X−H in which X is more electronegative than H, and an atom or a group of atoms in the same or a different molecule, in which there is evidence of bond formation”* (Arunan et al., 2011).

While hydrogen bonding may be quite recognizable, it can be difficult to define—and even harder to quantify (Weinhold and Klein, 2012). Steiner's (Steiner, 2002) review meticulously covered an extensive range of different hydrogen bond manifestations including the energy, charge density, spectroscopic properties, strength (strong, moderate, and weak), directionality (of both the donor and acceptor), and bond lengths. He concluded that the hydrogen bond is “*a complex interaction composed of several constituents that are different in their natures.”* This leads to hydrogen bonding affecting *all* components of the total interaction energy, and not simply electrostatics, induction, and exchange. Since hydrogen bonding is a very broad phenomenon, it cannot be strictly contained, but instead transitions and merges into other effects.

One of the quintessential examples of hydrogen bonding, the hydrogen bonded network in water, is largely driven by electrostatics and polarization effects (Lee and Rick, 2011). Other examples, such as Watson-Crick hydrogen bonding in DNA are electrostatic in nature (with significant charge transfer), and others may still be dispersion driven, such as the CH…π bond interactions in benzene crystals (Aida, 1988; Perutz, 1993). Hydrogen bond systems are traditionally defined as an interaction of the type: A…H−D, where H is the hydrogen atom, A is the acceptor, and D is the donor. H is bonded covalently to a donor atom, which is more electronegative than the hydrogen itself. This imbues hydrogen with a slight positive charge, which interacts attractively with the acceptor. According to Steiner, to qualify as a hydrogen bond, this interaction must fulfill two criteria (Steiner, 2002): (1) it is a local bond, and (2) D−H acts as a proton donor to A.

By these conditions, hydrogen bonding occurs in many ionic liquids, especially protic ionic liquids. The fields of both hydrogen bonding and ionic liquids are rich and complex, with significant overlap (Hunt et al., 2015). The characterization of hydrogen bonding in ionic liquids is by no means an easy task, with ionic liquids being a cocktail of manifold interactions. The medley of polar, nonpolar, organic, and inorganic constituents that can be incorporated into ionic liquids result in a complex representation from both electrostatic and dispersion forces present in these compounds, which have been studied from a range of computational viewpoints (Hunt et al., 2006; Bedrov et al., 2010). Dong et al. found that hydrogen bonding is a “major intermolecular structural feature” through DFT calculations of [C_2_mim][BF_4_] and [C_4_mim][PF_6_], examples of typical ionic liquids (Dong et al., 2012). Compared to protic ionic liquids, aprotic types are less likely to form directional hydrogen bonds (Stoimenovski et al., 2011). The C2-H bond, which is the most acidic proton on the imidazolium cation, is often involved in these non-directional hydrogen bonds. Conversely, protic ionic liquids, which are formed via a proton transfer reaction from a Brønsted acid to Brønsted base (Greaves et al., 2008; Simons et al., 2016), are more likely to form directional hydrogen bonds. This proton can subsequently be involved in a hydrogen bonding interaction with the anion. *Ab initio* molecular dynamics (AIMD) has been shown as necessary to identify these hydrogen bonds, as there are cases where classical molecular dynamics do not locate them (Maginn, 2007). For example, Del Pópolo, who was the first to run AIMD calculations for ionic liquids, used it to predict the structure of [C_1_mim]Cl and model the proton transfer from HCl. Strong in-plane hydrogen bonding with the C2-H bond has been observed in AIMD but not in classical molecular dynamics; further confirmed by Bühl et al. (2005), Del Pópolo et al. (2005), and Bhargava and Balasubramanian (2006).

Our recent review on quantum chemical methods for ionic liquids looked into the nature of hydrogen bonding within these semi-Coulombic systems in a great detail (Izgorodina et al., 2017). No significant energetic differences were found in a series of single ion pairs of aprotic ionic liquids consisting of imidazolium- and pyrrolidinium-based ionic liquids, with the former showing slightly larger dispersion components (Izgorodina and MacFarlane, 2011; Tan and Izgorodina, 2016). In terms of transport properties, the low viscosity of imidazolium ionic liquids has been largely attributed to the hydrogen bonding that occurs between the C2-H hydrogen and the anion (Fumino et al., 2009; Wulf et al., 2010; Dong et al., 2012). These studies have led to suggest that hydrogen bonding in ionic liquids might require a different treatment to typical cation-anion interactions when studied with classical force fields. This has motivated many to develop either different approaches to describing hydrogen bonding in ionic liquids in molecular dynamics force fields, or entirely new force fields solely for ionic liquids (Dommert et al., 2014; McDaniel et al., 2016). Protic ionic liquids in particular have linear hydrogen bonds as observed in their crystal structures (Henderson et al., 2012). Compared to aprotic ionic liquids, these directional hydrogen bonds also make them less viscous and result in higher dielectric constants (Weingärtner, 2014). Some of the hydrogen bonds can form extended hydrogen bonded networks, resulting in high melting points above 100 °C (Stoimenovski et al., 2010).

Regardless of the composition of ionic liquids, it has been established that the ions become strongly polarized in the bulk (Del Pópolo et al., 2005; Prado et al., 2006; Rigby and Izgorodina, 2013; Halat et al., 2017) and therefore, their geometry and energetics is strongly affected by the presence of neighboring ions (Wendler et al., 2011; Pensado et al., 2012). This study seeks to determine whether hydrogen bonding in ionic liquids results in interaction energies that differ from those of typical (i.e., non-specific) inter-ionic interactions. Single ion pairs of protic ionic liquids (PILs) were optimized in both the gas phase and implicit solvent and their energetic parameters were compared with those of archetypical ionic liquids based on imidazolium and pyrrolidinium cations. In addition, interaction energies of PILs in the gas phase and an implicit solvent were decomposed into electrostatic and dispersion components. Although the use of single ion pairs of hydrogen bonded ionic liquids cannot truly encapsulate the complexities of a bulk ionic liquid in which there would be multiple possible particle-particle interactions and various hydrogen bonded geometries, this article aims to provide a broad snapshot of several likely hydrogen bonding interactions that would be present in ionic liquids, and analyze their energetic components without claiming to represent all possible configurations that would be present in a bulk hydrogen bonded ionic liquid. Nevertheless, the obtained data provide an insight into the types of interactions within a hydrogen bonding ionic liquid and are used to comment on the debate whether hydrogen bonded ionic liquid requires special treatment. Equally important, we investigated the use of implicit solvent models to predict hydrogen-bonded structures of ionic liquids—as the gas phase tends to unrealistically reduce the hydrogen bond length between the cation and anion—thus rendering their energetic analysis possible.

## 2. Theoretical Procedures

To study a range of hydrogen bonded ionic liquids with varying ions, the HBIL dataset was created from the following sets of six cations and six anions: dimethylethylammonium (DMEA^+^), ethylmethylammonium (${\text{EtMeNH}}_{2}^{+}$), ethylammonium (${\text{EtNH}}_{3}^{+}$), trimethlyethylammonium (TMEA^+^), 1-methylimidazolium (mim^+^) and N-methylpyrrolidinium (mpyr^+^)—and anions: trifluoroacetate (CF_3_COO^–^), trifluoromethyl sulfonate (CF_3_${\text{SO}}_{3}^{–}$), methylsulfate (CH^3^${\text{SO}}_{3}^{–}$), methylsulfonate (${\text{MeOSO}}_{3}^{–}$), nitrate (${\text{NO}}_{3}^{–}$), and chloride (Cl^–^). All cations are protonated on the nitrogen center bases and were chosen as they form protic ionic liquids.

**Figure 1:**
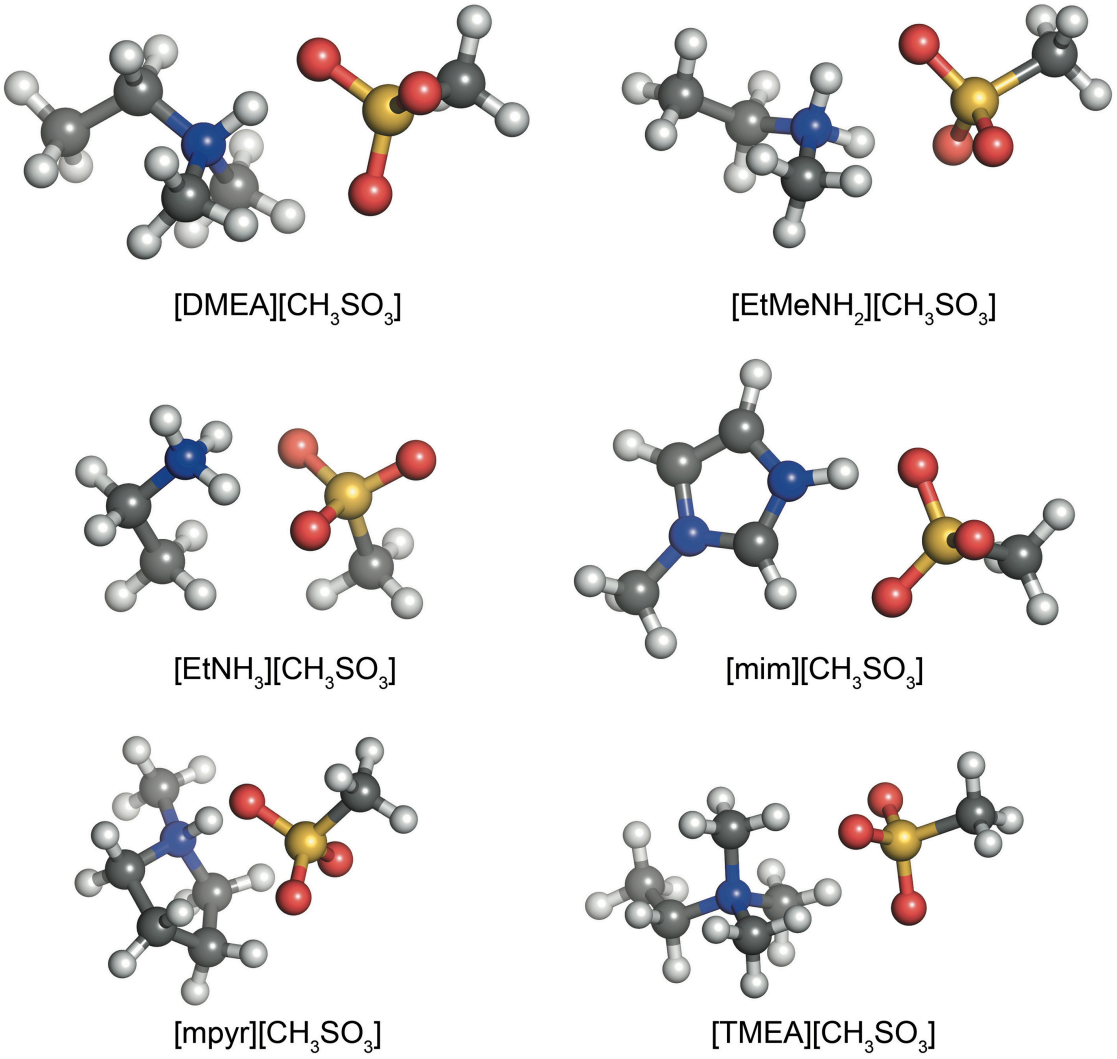
The various cations in the HBIL dataset, paired with the methylsuphonate anion.

Similar to the previously established IL174 dataset (Rigby and Izgorodina, 2014a), all systems here consist of combinations of a cation and an anion to form a single ion pair with a hydrogen bond between the N−H bond on the cation and an oxygen atom on the anion. Examples of these configurations are given in Figure 1 for the six different cations and the methylsulfonate anion. In the case of the mim^+^ and mpyr^+^ cations, multiple interaction sites are present on the cation (Izgorodina and MacFarlane, 2011; Izgorodina et al., 2011). For example, 1-methylimidazolium can interact with the C2-H bond in the plane of the ring, or above the ring. These configurations form a subset of the HBIL dataset, and their analysis is included separately from the rest of the HBIL set. Some combinations only have one configuration, such as in most chloride systems. All initial geometries were chosen to best reflect energetically preferred ion pair configurations observed in imidazolium- and pyrrolidinium-based ionic liquids (Izgorodina et al., 2014) and maximize the hydrogen bond interaction. In systems were both hydrogen bonded, and non-hydrogen bonded interactions were possible, multiple initial configurations were chosen and optimized. However, the scope of this article is to study several, and not all, possible configurations of single ion pair ionic liquids to give an insight into the different types of energetics and bonding of hydrogen bonded ionic liquids.

Geometry optimizations were performed using the M06-2X functional (Zhao and Truhlar, 2008) and the cc-pVTZ basis set, in both the gas phase and implicit solvent. Ground state geometries were confirmed by frequency calculations indicating no imaginary frequencies present. All optimized structures can be found in the Supplementary Material.

The Conductor-like Polarizable Continuum Model (CPCM) (Klamt and Schüürmann, 1993; Barone and Cossi, 1998) was used to model implicit solvent, which was represented by the dielectric constant of ethanol. Ethanol was chosen as the solvent as its dielectric constant is representative of ionic liquids, which usually fall between 10 and 16, and up to 25–50 for protic ionic liquids (Singh and Kumar, 2008; Weingärtner, 2008). Previously we have shown that ethanol was a good model of the ionic liquid bulk for geometry optimizations, (Chen and Izgorodina, 2017) producing already reliable geometries for single ion pairs of aprotic ionic liquids. Geometry optimizations were performed using the Gaussian09 software, and single-point energy calculations were carried out in PSI4 (Frisch et al., 2009; Turney et al., 2012). To calculate and decompose the total interaction energy into its electrostatic and dispersion components, first the electronic energy of the entire ion pair was calculated. The individual electronic energies of the cation and anion were then subtracted from the total energy, leaving behind only the energy of interaction between the cation and anion. Counterpoise-corrected Hartree-Fock energies (HF/aug-cc-pVQZ) energies were used to calculate the electrostatic contribution to the interaction energy, and Halkier's (Halkier et al., 1999) method of extrapolating to the complete basis set (CBS) was applied to MP2 and coupled-cluster results to calculate the correction to the correlation energy [MP2/CBS + ΔCCSD(T)]; giving the contribution from electron-correlated effects, i.e., dispersion. The Boys and Bernardi method were used to calculate counterpoise correction with HF, MP2, and CCSD(T) levels of theory (Boys and Bernardi, 1970). The total CCSD(T)/CBS interaction energy was calculated as follows:

(1) $$\begin{array}{l} E(CCSD(T)/CBS)=E_{HF/aug-cc-pVQZ}+E_{MP2/CBS}\\ \;+\Delta CCSD(T)/CBS \end{array}$$

The interaction energy is decomposed into two main components: electrostatic (represented by the HF energy) and dispersion (represented by the CCSD(T)/CBS correlation energy).

## 3. Results and Discussion

### 3.1. Gas and Solvent Phase Geometry Optimizations

Geometry optimizations are frequently done in the gas phase to avoid additional computational costs. For condensed systems—be it liquid or solid—this approach may result in geometries that differ from those found experimentally, such as through X-ray crystallography. This might be particularly evident for systems driven by electrostatic interactions such as protic ionic liquids. This is a result of the gas phase calculations not taking into consideration the stabilizing effect of the surrounding ions, thus producing shorter intermolecular distances (Chen and Izgorodina, 2017). Although this may not pose a problem if the focus of a study is on the *nature* of the intermolecular interaction and less on ideal geometries, there may be times where inadequate geometries are used for purposes that warrant higher accuracy. It is well-known that the gas phase destabilizes electrostatic interactions and therefore seeks to reduce them, usually by favoring the back proton transfer from the cation to the anion in protic ionic liquids, thus forming neutral species. The use of implicit solvent has been demonstrated to lead to more energetically stable complexes of predominantly ionic nature (Mackerell et al., 2004; Chesman et al., 2014). A well-known example is the preference of amino acids to adopt the zwitterionic form in aqueous media, whereas in the gas phase they are optimized to be neutral (Stover et al., 2012). Polar solvent molecules may interact more strongly with ions and thus shift the balance toward the formation of charged species. In the gas phase, this does not occur as no additional stabilization is available for charge-separated ions.

For the purposes of studying differences in geometries optimized in the gas phase and implicit solvent, the hydrogen bonded ionic liquids dataset (further in the text referred to as the HBIL dataset) were optimized under both conditions, using the same starting geometries. These were constructed with a linear hydrogen bond between the N-H bond on the cation and an electronegative atom (such as oxygen) on the anion. Some systems had more than one ion-pair configuration to reflect the different potential interactions possible, such as the chloride ion interacting either in-plane or above the plane of the imidazolium cation. The latter configuration does not have hydrogen bonding between the ions but is a known configuration in these systems (Izgorodina and MacFarlane, 2011).

**Figure 2:**
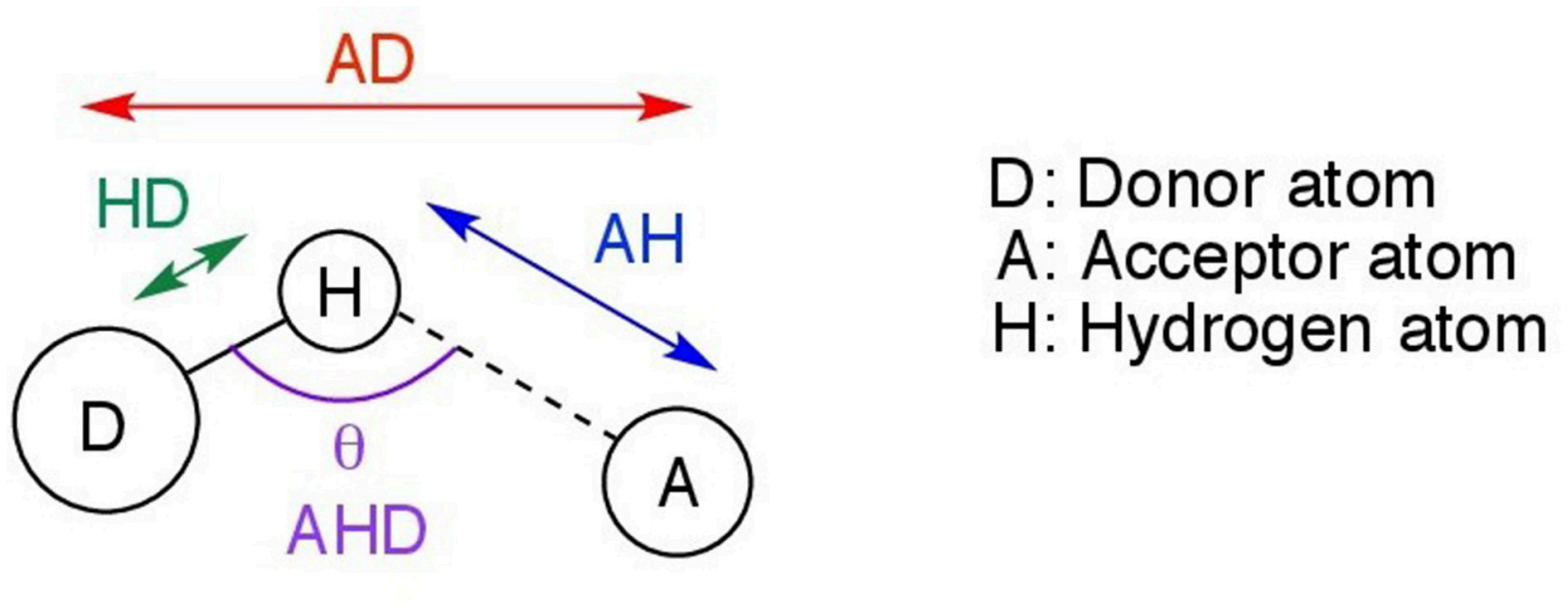
Hydrogen bonding parameters AD, AH, AHD, and HD measured in this study.

This section discusses the geometries resulting from both the gas phase and implicit solvent optimizations, their differences, and significance. Further in the text, the following notation is used. D denotes the donor atom (which “donates” the hydrogen to the electronegative atom), and A, the acceptor atom as shown in Figure 2. Possible acceptor atoms consisted of either oxygen or chlorine, and possible donor atoms were either carbon or nitrogen. A hydrogen bond system, A…H−D, is defined by any three of the following four parameters: the hydrogen bond distance (AH), the length of the hydrogen donor bond (HD), the distance from the acceptor to the donor (AD), and the hydrogen bond angle (AHD). All four predicted parameters are discussed below.

**Figure 3:**
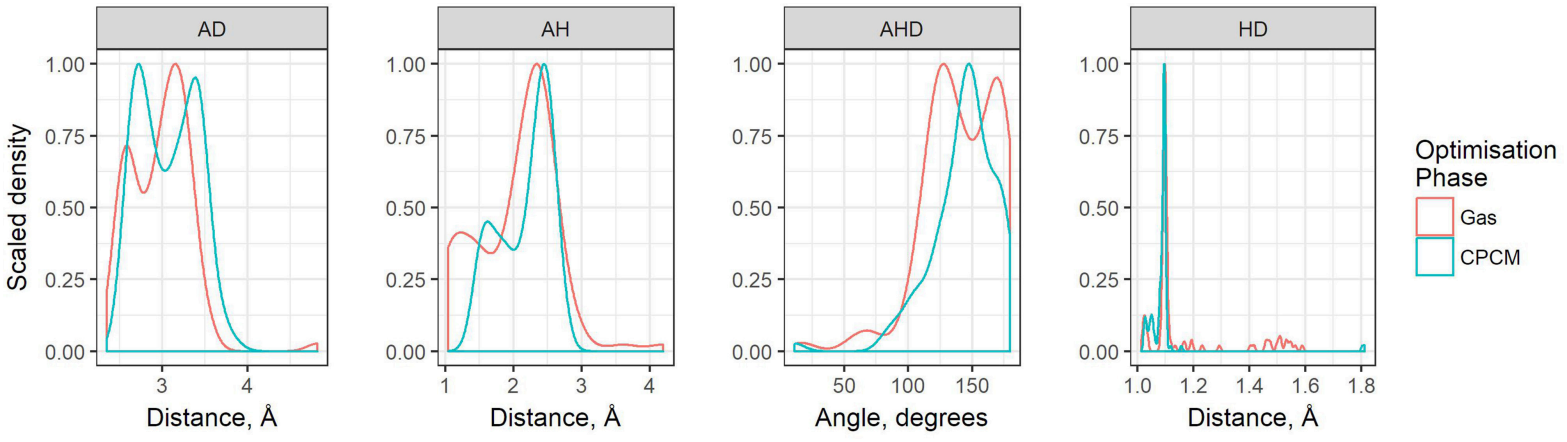
Scaled density plots of hydrogen bonding parameters AD, HD, AH, and AHD, measured for ion pairs in HBIL (solvent and gas phase optimization). Scaled density is calculated from *density value / max(density)* where *density = count / sum(counts)*.

**Figure 4:**
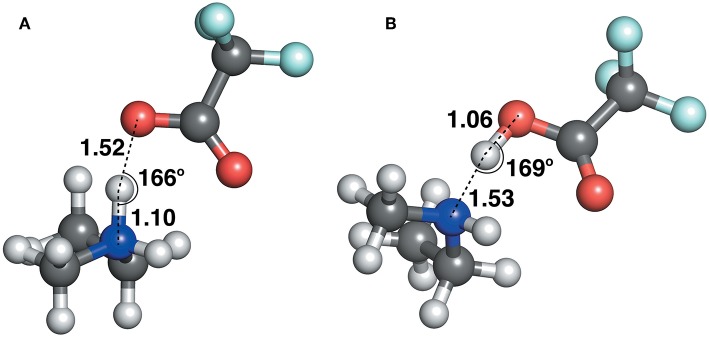
Ethylmethylammonium trifluoroacetate structure, optimized with **(A)** CPCM in ethanol (left) and **(B)** in the gas phase (right).

The geometric parameters obtained from both modes of optimization is plotted as scaled density plots in Figure 3. For the acceptor-donor distance (AD), both distributions are similar in shape, with gas-phase distances shifted to shorter numbers by 1.3 Å on average. This is also seen in the distributions for the hydrogen bond distances, AH. These distances may be up to 0.7 Å longer in implicit solvent for methylpyrrolidinium-based ionic liquids; due to back proton transfer occurring from the anion to cation when optimized in the gas phase (see Figure 4). The second peak in the distribution for AH relates to ionic liquids, for which the back-proton transfer has not occurred in the gas phase. AH tends to be shorter by an average of only 0.1 Å compared to implicit solvent, with good agreement for the methylimidazolium and pyrrolidinium-based cations in particular. Interestingly, for the hydrogen bond angle, AHD, implicit solvent has a peak at 150°, whereas the gas phase distribution is split into two peaks on either side of the solvent one. This suggests that gas phase optimizations tend to be more “extreme,” resolving systems into either clearly hydrogen bonded, or not, whereas implicit solvent tends to produce a structure in between. The distance between the hydrogen atom and a donor atom, HD, is a parameter which highlights the difference between the two different optimization modes. While implicit solvent bond lengths fall in a very narrow range, around 1.1 Å, there are several instances of the gas phase HD lengths which vary as much as 1.8 Å. In fact, there are cases optimized in the gas phase where the hydrogen bond length is much longer than that in implicit solvent. One such example is ethylmethylammonium trifluoroacetate (Figure 4), whose AH length was found to be 1.06 Å and the HD length—1.53 Å. In implicit solvent, these distances are 1.52 and 1.10 Å respectively. Back proton transfer occurred during the gas phase optimization, thus producing neutral species and disfavoring the formation of a protic ionic liquid. The anions that are more likely to result in the back-proton transfer during gas-phase optimizations are chloride, methylsulfate, trifluoromethylsulfonate, and in some cases trifluoroacetate.

In terms of differences between the gas phase and implicit solvent geometric parameters, gas phase optimizations tend to give shorter AD distances in the majority of cases, though there are some instances where AD is overestimated by more than 0.5 Å. Similarly, the AH distance becomes longer when a solvent model is applied. On the other hand, the HD bond length has smaller deviations relative to the other parameters due to the covalent nature of either the N−H bond or the C−H bond in the case of either TMEA or mim^+^ cations. This is the reason for why optimizations usually agree on the predicted bond length (except in cases where back proton transfer occurs). For the hydrogen bond angle, agreement between gas and solvent optimizations are good when the angles are nearly linear (around 175°). However, when implicit solvent optimizations predicted lower bond angles around 150°, gas phase optimizations both over- and under-estimated bond angles. Overestimation tends to happen more for nitrogen donors, though there are exceptions such as [EtMeNH_2_][MeOSO_3_], which has an angle of 158° in implicit solvent and 123° in gas phase.

**Figure 5:**
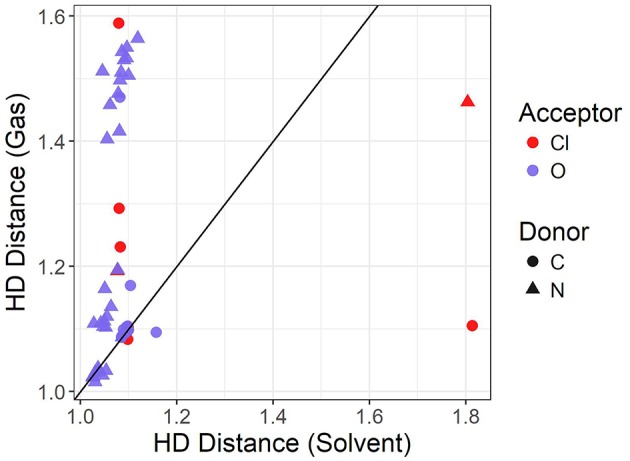
Solvent vs. gas phase optimization for the hydrogen-donor bond distance in Å.

The graph shown in Figure 5 compares the donor-hydrogen HD bond distance between the gas phase and implicit solvent optimizations. The gas phase parameters are plotted on the vertical axis, whereas the implicit solvent ones on the horizontal axis. Points are colored by the element of the acceptor atom and the shape of the point indicates the element of the donor. The diagonal black line is added as a visual guide only. The better the agreement between the two optimization modes, the closer to the diagonal line the predicted points should fall. As can be seen, two clusters are delineated by the gas phase distances. Implicit solvent bond lengths have a very narrow range, with the vast majority falling between 1 and 1.2 Å, due to the proton transfer from the acid to the base being strongly favored. There are a few outliers involving hydrogen bonds with the chloride anion which have a typical distance of 1.8 Å: this occurs for the bulky quaternary ammonium cation TMEA, as well as the EtNH^3^ cation. In these cases, the longer bond length indicates the relatively weaker hydrogen-bonding interaction between the chloride and primary or quaternary amine, compared to the secondary amine or imidazolium/pyrrolidinium-based cations.

When an oxygen acceptor is involved (e.g., methylsulfate or nitrate), there is remarkable agreement on the C−H bond length between both optimization modes, with most of its points being tightly clustered on the line. This is expected, as it is usually understood to be a covalent bond. On the other hand, when the hydrogen is attached to a nitrogen donor, the points appear to follow a different trend. They agree for shorter distances, probably due to weaker interactions with the acceptor atom. Gas phase optimizations clearly struggle when the inter-ionic interaction becomes stronger, leading to instances for which the back-proton transfer occurs and hence no anion is formed. Examples of back-protonation include 1-methylimidazolium chloride and dimethylethylammonium when coupled with CH_3_${\text{SO}}_{3}^{–}$, ${\text{SO}}_{3}^{–}$, TFA, and similarly for 1-methylpyrollidinium with ${\text{NO}}_{3}^{–}$ and TFA.

#### 3.1.1. Comparison With Crystal Structures of Similar Ionic Liquids

While data for the particular systems in HBIL have not yet been obtained, it is still insightful to compare experimental data from crystal structures of similar ionic liquids with solvent and gas phase calculations. These can be used to provide a sense of the typical hydrogen bond length in these ionic liquids.

Table 1 presents some available experimental hydrogen bond parameters. At least two interactions are shown for each ionic liquid. Comparing the typical distances and angles obtained through geometry optimizations with those obtained from experimental crystal structures, experimental values fall in a relatively narrow range. For example, the hydrogen bond distance (as defined by AH) ranges from 1.6 to 2.7 Å. All four hydrogen bonding parameters from both optimization modes are shown in Table 2. Comparing the HD distance from geometry optimizations, the gas phase bond lengths are longer, except for the N…Cl donor-acceptor pair. The shorter bond distances for N…O and C…O systems from implicit solvent optimizations agree better with the experimental distances than gas optimizations. Most experimental data indicate that (all but 4) N−H or C−H distances fell below 1 Å, with the longest distance being 1.19 Å. In the optimized HBILs, the longest C−H bond with an O acceptor is 1.2 Å for both gas and CPCM optimizations. Similarly for the N−H bond, the longest distance in implicit solvent is found to be 1.1 or 1.2 Å in the gas phase, showing that both optimization modes have good agreement with experiment for this particular parameter. However, for the hydrogen bond distance parameter, AH, in the C…O systems, the range of experimental distances (1.9–2.6 Å) is much closer to the implicit solvent values (1.5–2.7 Å) compared to the gas phase values (1.5–4.2 Å). For N…O donor-acceptor pairs, the hydrogen bond is stronger than the systems with a carbon donor. This is reflected in the shorter average bond lengths, ranging from 1.6 to 2.5 Å. This is also observed in both optimization modes, but again implicit solvent optimizations (1.5–2.5 Å) tend to fare better than the gas phase ones (1.3–3.6 Å).

**Table 1:** Hydrogen bonding parameters taken from experimental ionic liquid crystal structures.

**Ionic liquid**	**Donor**	**Acceptor**	**HD**	**AH**	**AD**	**AHD**
1-(2-hydroxyethyl) yrrolidine-1-ium	N	O	1.06	1.61	2.651	165
benzoate	C	O	0.99	2.53	3.382	145
1-(2-hydroxy-ethyl)pyrrolidinium	N	O	0.99	2.50	3.418	153
2,5-dihydroxy-benzoate	C	O	0.99	1.91	2.778	162
1-(2-hydroxyethyl) yrrolidine-1-ium	N	O	0.99	1.81	2.697	169
2-hydroxy-benzoate	C	O	0.99	2.51	3.391	148
(3s,5s,7s)-adamantan-1-ammonium	N	O	0.99	1.80	2.777	170
benzoate	N	O	1.19	1.60	2.278	169
	C	O	0.95	2.42	2.749	100
(3s,5s,7s)-adamantan-1-ammonium	N	O	1.03	1.76	2.781	170
2,5-dihydroxy-benzoate	N	O	0.91	1.93	2.809	162
(3s,5s,7s)-adamantan-1-ammonium	N	O	0.93	1.90	2.814	168
2-hydroxybenzoate	N	O	1.01	1.85	2.825	162
	C	O	0.97	2.58	3.440	148
heptan-2-ammonium	O	O	0.98	1.70	2.662	167
2,5-dihydroxy-benzoate	N	O	0.97	1.81	2.777	173
	O	O	0.82	1.77	2.544	157
	N	O	0.98	1.90	2.848	163
	N	O	0.87	2.42	2.979	122
	N	O	0.87	2.27	2.905	130
	C	O	0.95	2.57	3.225	126
butyl dimethyl imidazolium	C	O		2.41	3.360	147
hydrogen sulphate	C	O		2.45	3.230	166
butyl dimethyl imidazolium	C	Cl		2.68		157
chloride	C	Cl		2.59		168

*Benzoate ionic liquids from Stoimenovski et al. (2011), and butyl dimenthyl imidazolium structures from Kölle and Dronskowski (2004)*.

**Table 2:** Hydrogen bond parameters by donor acceptor pairs for the gas phase and implicit solvent optimization modes.

**Phase**	**Donor**	**Acceptor**	**MAE**	**SD**	**Min**	**Max**	**MAE**	**SD**	**Min**	**Max**
			**HD Distance**				**HA Distance**			
CPCM	C	Cl	1.181	0.255	1.080	1.813	2.294	0.360	1.866	2.667
Gas	C	Cl	1.146	0.082	1.084	1.293	2.204	0.466	1.536	2.747
CPCM	N	Cl	1.440	0.514	1.077	1.803	1.896	0.010	1.889	1.903
Gas	N	Cl	1.193				1.631			
CPCM	C	O	1.096	0.009	1.082	1.157	2.373	0.230	1.539	2.703
Gas	C	O	1.099	0.011	1.087	1.170	2.335	0.354	1.529	4.196
CPCM	N	O	1.059	0.026	1.025	1.119	1.811	0.323	1.454	2.547
Gas	N	O	1.075	0.055	1.015	1.195	1.989	0.613	1.304	3.598
			**DA Distance**				**HBA Angle**			
CPCM	C	Cl	3.358	0.364	2.948	3.829	160.6	11.7	151.4	178.4
Gas	C	Cl	3.031	0.382	2.349	3.358	137.2	37.2	67.4	178.8
CPCM	N	Cl	2.969	0.007	2.964	2.974	172.5	4.7	169.2	175.8
Gas	N	Cl	2.822				176.5			
CPCM	C	O	3.237	0.237	2.628	3.646	138.7	19.6	81.3	177.9
Gas	C	O	3.186	0.278	2.695	4.819	134.0	23.7	16.5	175.8
CPCM	N	O	2.705	0.096	2.556	2.950	145.3	35.2	11.4	178.4
Gas	N	O	2.718	0.244	2.495	3.472	134.8	35.9	57.8	178.8

*All distances are given in Å*.

Experimental donor-acceptor distances are only available for the C…O and N…O systems. For the C…O cases, experimental values range from 2.7 to 3.4 Å which is closely reflected in the range of implicit solvent donor-acceptor distances of 2.6–3.6 Å. Gas phase has a higher maximum value, ranging from 2.7 to 4.8 Å. Once again, the experimental DA distances for the N…O systems are shorter and closer to the implicit solvent ones.

Hydrogen bond angles are an important indicator of hydrogen bonding, since directionality is a well-recognized characteristic of the interaction. For C…Cl systems, the implicit solvent average of 160° is much closer to the angles seen in experimental data (157° and 168°), compared to the gas phase average of 137°. For C…O systems, experimental angles range from 100° to 166°. While both optimization modes have similar averages, the implicit solvent values are closer to experiment (81°–178°), compared to the gas phase ones which unsurprisingly contain more outliers in the range of (17°–176°). Systems with nitrogen as a donor and oxygen as an acceptor show characteristics of a stronger hydrogen bond, with experimental data having higher angles, ranging from 122° to 173°. The gas phase seems to be a poor fit overall, ranging from 58 to 179° with an average of 135°. However, implicit solvent values did not perform much better, ranging from 11 to 178°, with a slightly higher average of 145°.

Overall, it is not too surprising that these results indicate that an implicit solvent model is necessary when performing geometry optimizations involving polar interactions such as hydrogen bonding. The CPCM model clearly stabilizes the charges on the ionic species, leading to more relaxed geometries. Gas phase geometries often result in shorter separations, which may not be a major issue if the goal is to qualitatively understand possible interaction configurations. However, there are many systems, including ionic liquids, where configurations exist only due to stabilizing effects from the environment. The use of implicit solvent models can help to replicate these effects and locate more accurate geometries without the need to perform large-scale calculations.

### 3.2. Energetic Differences in Hydrogen Bonded Ionic Liquids

**Figure 6:**
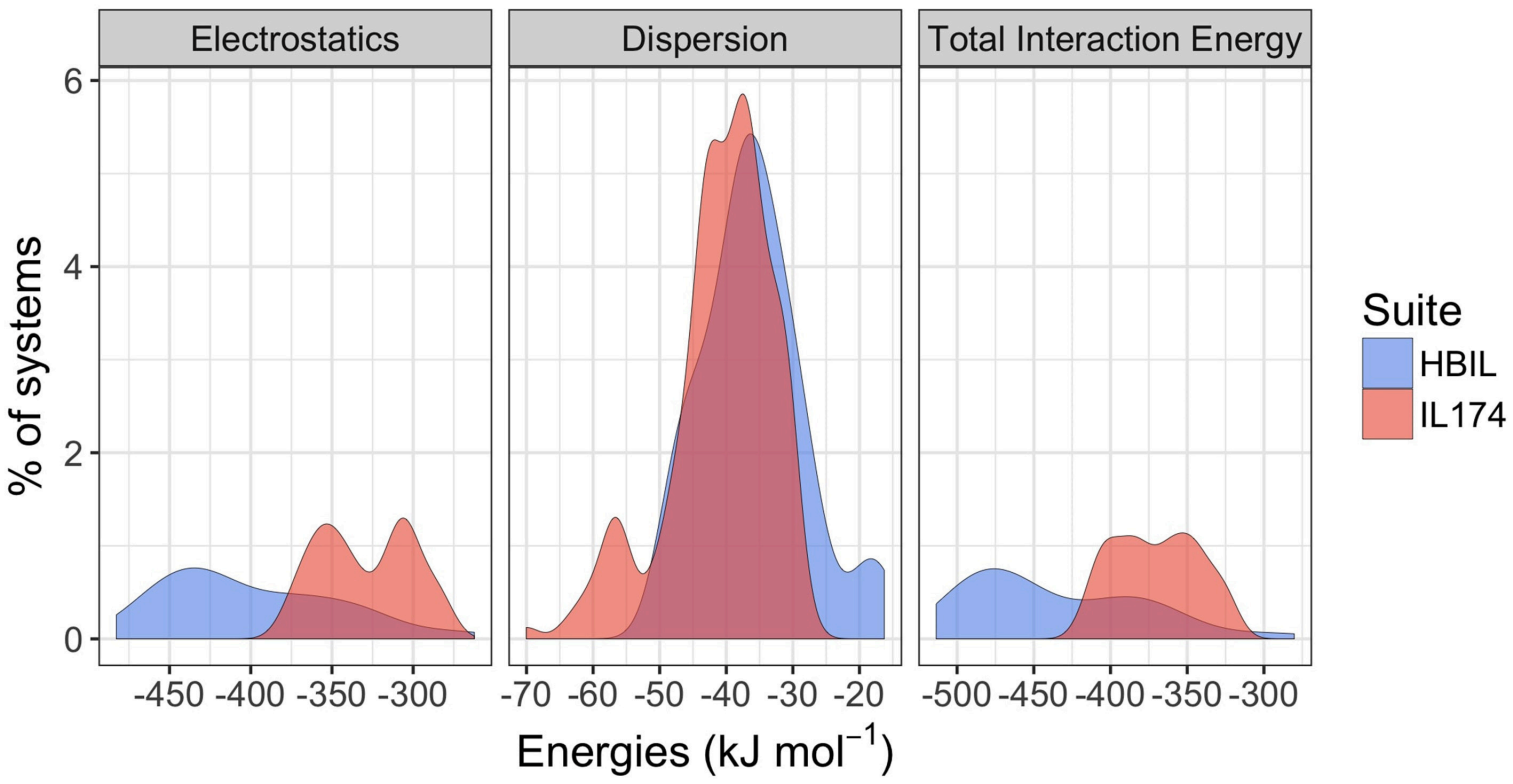
Comparison of gas-phase interaction energy distributions between the IL174 and HBILs datasets.

As one of the major goals of this work was to determine whether it is possible to identify and characterize hydrogen bonding in protic ionic liquids by its energy decomposition, interaction energy calculations were performed for the HBILs and compared with available data for the IL174 dataset, a dataset consisting of 174 single ion pairs of (mostly non-hydrogen bonding) aprotic ionic liquids (Rigby and Izgorodina, 2014b) based on imidazolium and pyrrolidinium cations and widely used anions. Full interaction energy data are available in the Supplementary Material. Only the solvent-optimized HBIL structures are included in the energetic calculations. The two components of total interaction energy of both datasets are plotted as distributions in Figure 6. The vertical axis represents a relative (in percentage) count for each energy value. Distributions that have a larger range, such as electrostatics in HBILs, seem smaller, but the area under the curves still represents the total percentage of systems.

Immediately noticeable is how significant the electrostatic energies are for hydrogen bonded ionic liquids. This is to be expected, as the nature of hydrogen bonding is predominantly electrostatic and polarizable in nature (Stoimenovski et al., 2011). This therefore translates to higher electrostatic energies and hence, stronger total interaction energies. For HBILs, electrostatic contributions range between −482.7 to −262.3 kJ mol^−1^, and dispersion between −49.5 and −16.3 kJ mol^−1^. The latter is more comparable with that of archetypical ionic liquids for which the dispersion contribution is between −70.0 and −27.5 kJ mol^−1^. Probably the most striking observation is how the total interaction energy can span a very wide range of 230 kJ mol^−1^ in hydrogen-bonded ionic liquids, whereas archetypical ionic liquids tend to have their total interaction energy clustered around the −370.1 kJ mol^−1^ value. This is at least 75 kJ mol^−1^ lower in energy than the average value for the HBILs, attributed predominantly to the electrostatic component. To complement Figure 6, the average, standard deviation, minimum and maximum values of each energetic component is shown in Table 3. Comparison of interaction energies between the gas phase and implicit solvent geometries is also included in Table 4.

**Table 3:** Comparison of interaction energy statistics (kJ mol^−1^) for the HBIL (implicit solvent optimization) and IL174 datasets.

**Dataset**	**Energy type**	**Mean**	**Std Dev**	**Min**	**Max**
HBIL	Electrostatics	−399.3	±53.0	−482.7	−262.3
	Dispersion	−36.0	±7.8	−49.5	−16.3
	Total	−435.2	±56.6	−513.6	−280.3
IL174	Electrostatics	−329.2	±28.3	−378.7	−278.1
	Dispersion	−40.9	±7.9	−70.0	−27.5
	Total	−370.1	±27.2	−417.7	−319.3

**Table 4:** Interaction energy statistics (kJ mol^−1^) for the HBIL dataset for gas phase optimization.

**Phase**	**Energy type**	**Mean**	**Std Dev**	**Min**	**Max**
Gas	Electrostatics	−301.0	±184.4	−730.9	−34.9
	Dispersion	−39.9	±10.8	−55.9	−16.3
	Total	−340.0	±191.4	−760.3	−56.3

These numbers once again highlight the discrepancy in interaction energy results when failing to include an implicit solvent model when optimizing single ion pairs of ionic liquids. The energetics of the gas phase structures have a much larger range for total energy, between −56.4 and −760.3 kJ mol^−1^. These values present both extremes of the spectrum, with the close to zero interaction energy indicating that the ions have little attractive force between them, which would naturally occur in cases when back proton transfer takes place and there are two neutral species. Conversely, the several hundred kJ mol^−1^ interaction energy occurs in gas phase by bringing the ions closer together than what is representative of reality, due to there being no solvent present to offset some of the attractive charge. To illustrate, the system with the largest interaction energy in gas phase, [EtMeNH_2_][TFA] (–760.3 kJ mol^−1^), has its value of interaction energy reduced by almost a half (–492.4 kJ mol^−1^) when a solvent model is used during optimization. Back-protonation occurs in the gas-optimized system, bringing the ions in close proximity. On the other hand, for system where back-protonation does not occur, [DMEA][CH_3_OSO_3_], interaction energy values are relatively more in agreement between the gas (–480.6 kJ mol^−1^) and solvent (–455.1 kJ mol^−1^) phases.

**Figure 7:**
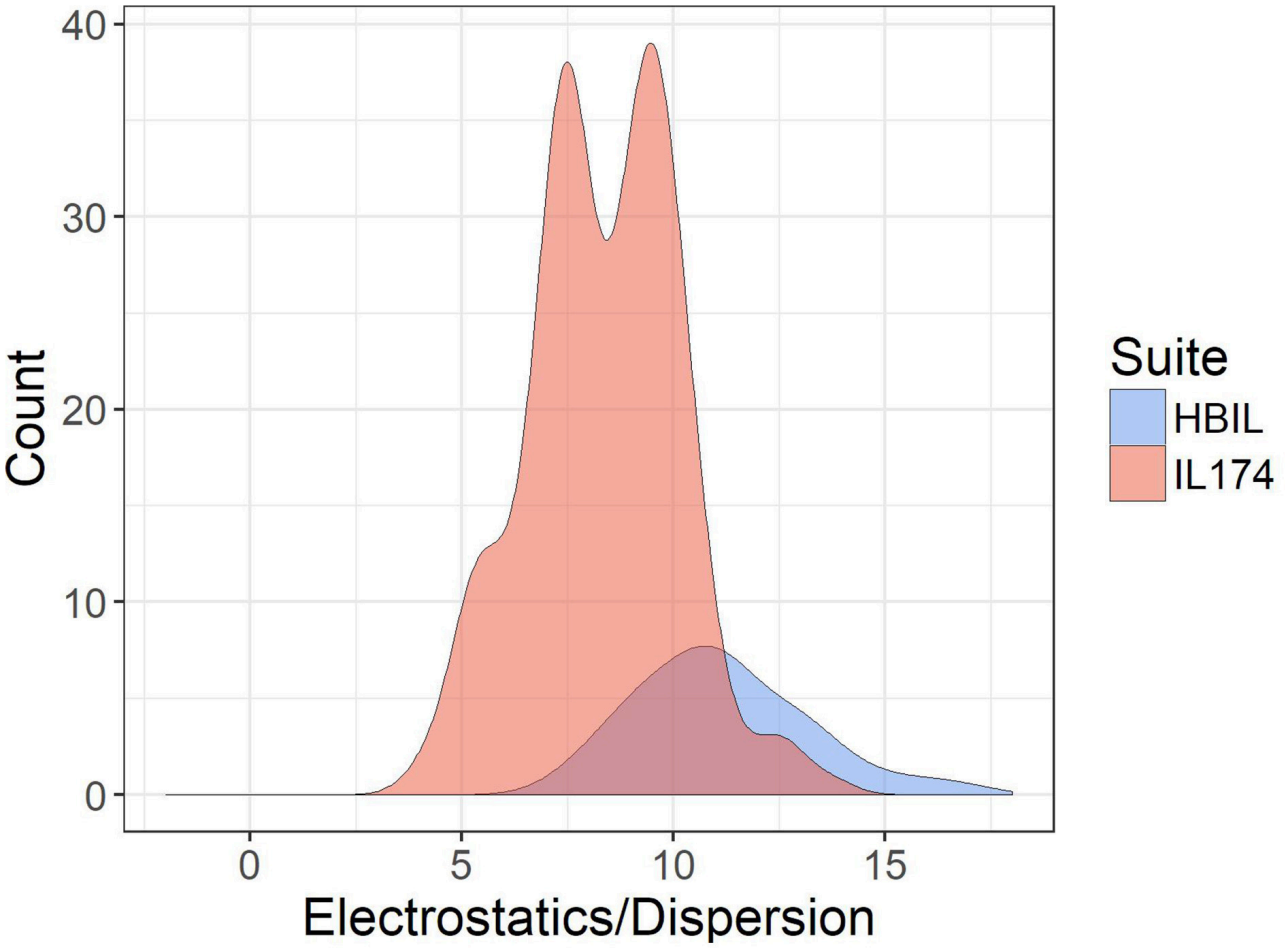
Ratio of electrostatic to dispersion energies for the IL174 and HBIL datasets. All interaction energies were calculated in the gas phase.

The ratio of electrostatic interaction energy is compared with dispersion energy in Figure 7. The majority of HBILs have a broad distribution of electrostatic interaction energy falling between 5 and 15 times the magnitude of the dispersion energy. This is similar to the IL174 dataset, for which the electrostatic interaction energy is clustered in the range of 5–10 times the dispersion energy.

**Figure 8:**
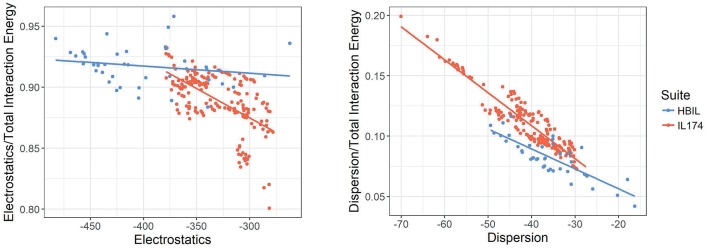
Electrostatics **(left)** and dispersion **(right)** against their relative contribution to the total gas-phase interaction energy for IL174 and HBIL datasets in kJ mol^−1^.

In the following discussion, the relative energy of each component is discussed. This is defined as the energy of the component divided by the total interaction energy. Figure 8 plots the electrostatic interaction energy and dispersion energy against their contribution to the total interaction energy. While IL174 ionic liquids tend to be clustered in one area, its slope is sharper, indicating that the contributions from electrostatics decreases with increasing dispersion. Contrary to this trend, HBILs tend to maintain a relatively constant electrostatic contribution of about 92% to the total interaction energy. The relative contribution from the dispersion energy (Figure 8, right) shows a clear difference between the HBIL and IL174 datasets. The gains in the total interaction energy from hydrogen bonding do not come from the dispersion component, hence lower contributions are seen for the HBILs, with the majority falling around 10%.

Plots depicting components of the interaction energy and their relative contributions all reflect the same trends: hydrogen bonding leads to stronger interactions, but these increases do not come from one component alone. Determining whether a system is hydrogen bonded based on its energetics does not seem to be a reliable method in this implementation. While hydrogen bonded ionic liquids do follow different energetic trends, it could be difficult to decide which category a system falls into since there is a major overlap in the energy distributions. Thus far, the best indicator to detect hydrogen bonding in ionic liquids is to compare the electrostatic and dispersion energies. The dispersion component is largely unaffected by hydrogen bonding, whereas the electrostatic components are usually larger. Comparing the differences or ratios of the electrostatic and dispersion energies appears to be a good, though not completely bulletproof, method to determine hydrogen bonding in an ionic liquid system.

### 3.3. Relationship Between Energies and Geometries

The final part of the results considers the relationship between the geometric parameters for hydrogen bonding and interaction energy decomposition.

**Figure 9:**
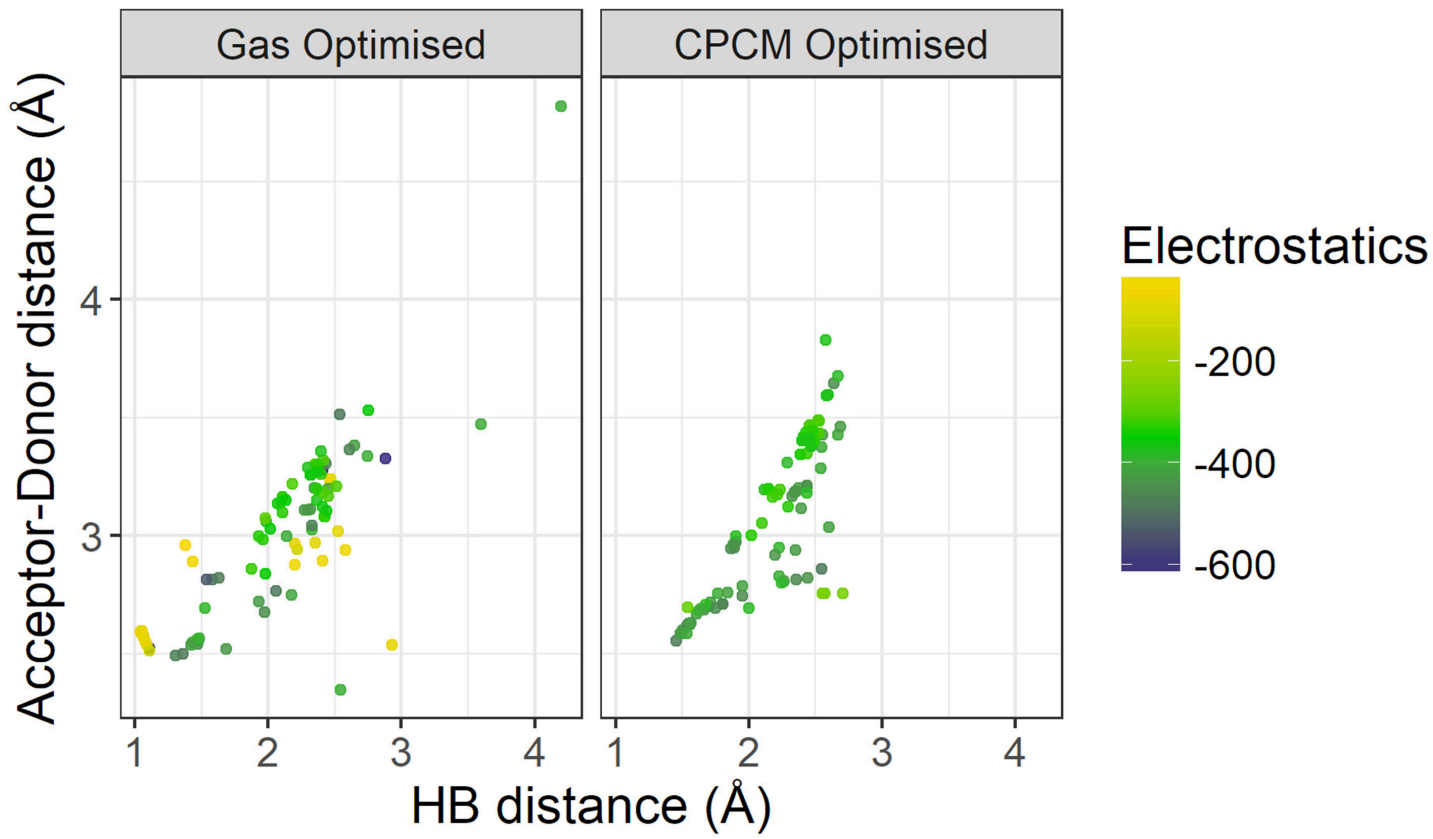
Comparison of Acceptor-Donor distance against hydrogen bond distances for gas phase and implicit solvent optimized geometries, colored by strength of electrostatics.

**Figure 10:**
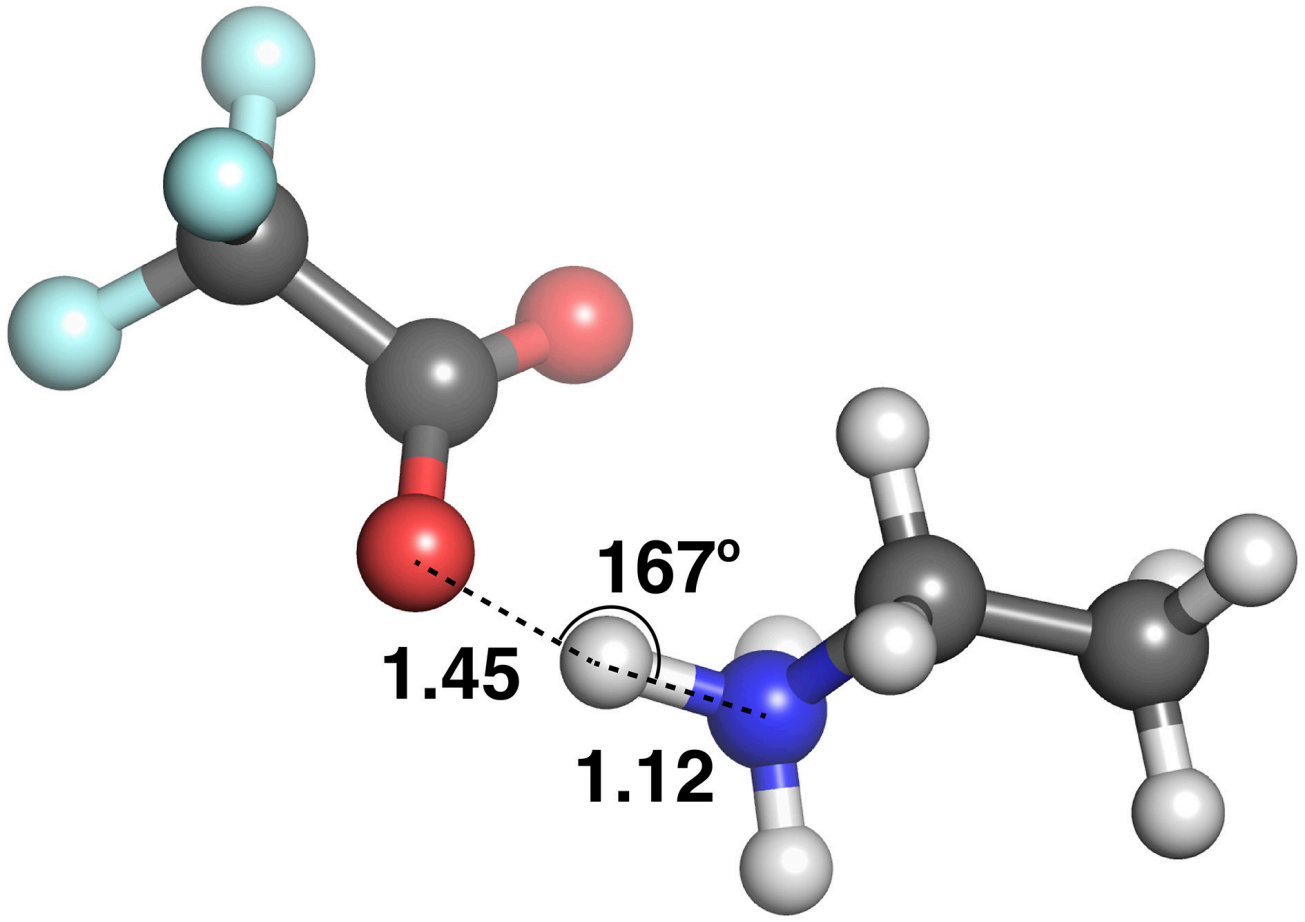
Ethylammonium trifluoroacetate, optimized with the CPCM model.

Figure 9 plots the distance between the acceptor and donor atoms against the hydrogen bond distance of the HBILs optimized in gas phase and implicit solvent. Points are colored by the strength of electrostatic interaction energy. Generally, the points follow a linear trend, which is expected as the electrostatic interaction is governed by the Coulomb's law confirming that an increase in the hydrogen bond length corresponds linearly to an increase in the acceptor-donor distance. The predicted points that do not follow this linear trend have bond angles that deviate significantly from 180°, often falling below 160°. The electrostatic interaction energy is higher for systems that are clearly hydrogen bonded, i.e., those with lower distances presented in the lower left corner of the graph. As the distance increases, the electrostatic interaction energy also decreases, due to a weakening interaction. However, this trend is not without exceptions. Several systems at longer separations, and indeed, non-linear bond angles, have strong electrostatic interaction energies. These systems include [EtNH_3_][TFA], which has a bond angle of 167° and is shown in Figure 10. Other examples include DMEA coupled with ${\text{MeSO}}_{3}^{–}$ and TFA, [EtMeNH_2_][TFA], ${\text{EtNH}}_{3}^{+}$ with ${\text{NO}}_{3}^{–}$ and TFA^–^. Notably, these all have SO_3_ or NO_3_ groups, electronegative groups that are able to form a strong hydrogen bond. This reflects the complicated nature of hydrogen bonded ionic liquids, where non-conventional lengths and angles can still result in significant electrostatic interactions.

On the left hand side of Figure 9, a comparison of the energetics of the implicit solvent optimized structures with those optimized in gas phase clearly highlights the failings of gas phase optimizations to capture the energetics of hydrogen bonding. There are a number of points with very weak electrostatic interaction energy (depicted in light yellow-green color) and are representative of structures for which the back-proton transfer occurs to form a neutral species. Gas-phase optimized structures clearly have weaker electrostatics compared to that of implicit solvent optimized ones, further highlighting the reduction of electrostatic interactions by gas phase in the absence of the stabilizing field of neighboring ions.

#### 3.3.1. Difference Between Gas and Solvent Optimizations and Total Interaction Energy

In this section, the hydrogen bond parameters from the gas phase and implicit solvent geometry optimizations are contrasted, while examining the strength of the electrostatic interaction in the two optimization modes. In the following plots, the shape of the point denotes the four possible donor-acceptor pairings: Cl−C, O−C, Cl−N, and O−N. The total interaction energy is chosen as it can be easily calculated using a variety of methods, and since electrostatics is the largest component of the total interaction energy (as shown previously), this hopefully means that any effect that hydrogen bonding has on electrostatics will be reflected in the total energy.

**Figure 11:**
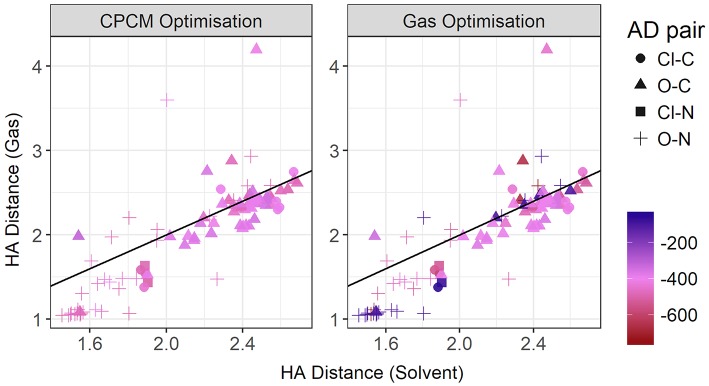
Gas vs. solvent optimized hydrogen bond distance in Angstrom, colored by strength of the total CPCM interaction energy **(left)**, and the total gas interaction energy **(right)**.

Figure 11 contrasts the HA distances observed in gas phase and implicit solvent. If we take the total energy as indicative of the hydrogen bond strength, then at shorter distances there is a strong correlation. These shorter distances are clearly hydrogen bonds in the conventional sense. It is noteworthy that most, if not all, are formed with nitrogen as the donor atom. As highlighted above, gas phase optimizations tend to underestimate these distances. However, at longer distances, solvent and gas phase optimizations agree better, with the gas phase optimizations tending to produce a wider range of HA distances > 3 Å, particularly for the sulphate-containing ionic liquids such as [DMEA][CF_3_SO_3_] and [DMEA][CH_3_SO_2_], [mim][CH_3_SO_3_], and [EtNH_3_][CH_3_OSO_3_]. These points are seen to lie closer to the diagonal line, likely due to the diffusive nature of these sulfur-containing anions compared to the others chosen such as TFA. Naturally, the stronger charge-localized ions are more likely to have shorter HA distances in the gas phase.

**Figure 12:**
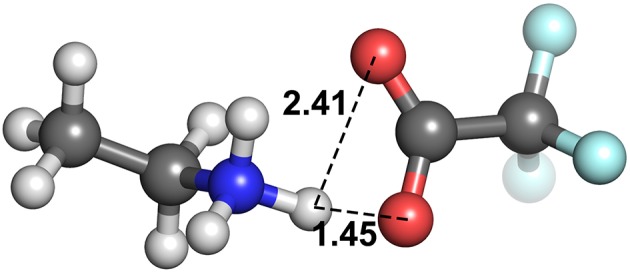
The two hydrogen bonds in [EtNH_3_][TFA](CPCMoptimized).

At these longer distances, there are also some systems with strong electrostatic interactions. In certain cases, there is more than one interaction occurring. For example, in [EtNH_3_][TFA], there is a clear hydrogen bond of 1.45 Å, and also another interaction involving the second oxygen of the anion and the same hydrogen, measuring at 2.4 Å (see Figure 12). The latter interaction is evidently not as strong; however due to the energy calculation encompassing the entire system, the resulting point appears to have a high energy even at a longer separation. A further analysis of these exceptions reveals that a few of these come from hydrogens attached to a carbon atom, and some of them from those attached to a nitrogen. This effect becomes very pronounced in the presence of multiple oxygen atoms on the anions such as TFA, CH_3_${\text{SO}}_{3}^{–}$, ${\text{NO}}_{3}^{–}$, interacting with other hydrogen atoms around a hydrogen bonds that is already short and strong, for primary and secondary amine cations ${\text{EtNH}}_{3}^{+}$ and ${\text{EtMeNH}}_{2}^{+}$ where there are multiple hydrogens available for hydrogen bonding. In regard to other cations such as imidazolium and pyrrolidinium, there is only one acidic proton available for interaction. This explains why several of the carbon donor systems have stronger Coulomb interactions, while occurring at longer separations. In general, carbon donor systems tend to form much weaker hydrogen bonds with energies between −300 and −350 kJ mol^−1^. In contrast, it is remarkable that all of the systems involving nitrogen as a donor have fairly strong electrostatics.

**Figure 13:**
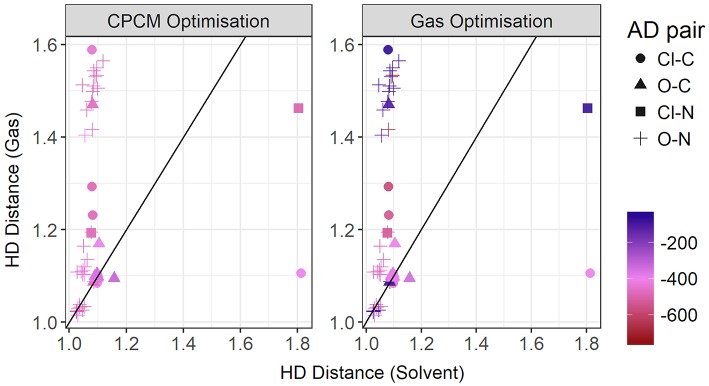
Gas vs. solvent optimized hydrogen-Donor distance in Å, colored by strength of the total CPCM interaction energy **(left)**, and the total gas interaction energy **(right)**.

In Figure 13, the gas phase optimized HD bond lengths are plotted against the implicit solvent ones. This is typically a covalent bond, however in gas phase optimizations it can sometimes be noticeably long. In some cases, it tends to be longer than a typical hydrogen bond itself. As discussed above, this is due to a number of systems undergoing the back-proton transfer. Only a small number of systems have agreement from both optimization modes. This includes all of the systems with weaker interactions, *i.e.*, having carbon donors and long hydrogen bonds as discussed previously. However, a significant number of hydrogen bonding systems, judging from both shorter bond lengths and stronger interaction energies observed in implicit solvent geometries, are relegated to longer bond lengths in gas phase due to the back-proton transfer. The nature of the acceptor atom seems to play a crucial role in determining whether gas phase geometries are adequate. It has to noted that the majority of the ionic liquids for which gas phase results favor neutral species, exist predominantly in their ionic form, such as [EtNH_3_][TFA] and [EtNH_3_][NO_3_] which have been experimentally observed to exhibit a significant extent of ionization. (Capelo et al., 2012; Greaves and Drummond, 2015).

**Figure 14:**
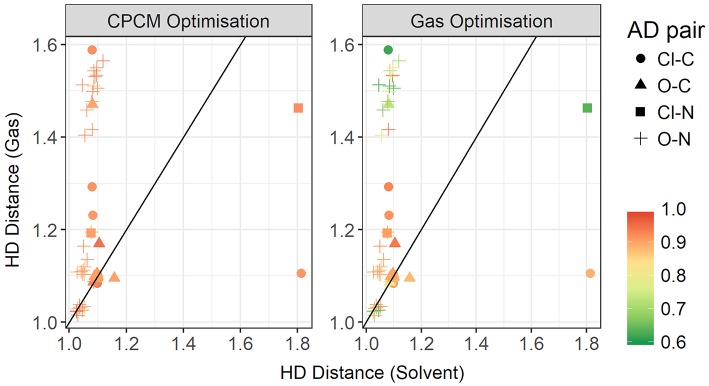
Gas vs. solvent optimized hydrogen-Donor distance in Å, colored by ratio of the electrostatic contribution to total CPCM interaction energy **(left)**, and the total gas interaction energy **(right)**.

To further investigate the relationship between the interaction energy and the hydrogen bond parameters, additional plots were made, this time colored by the *relative* electrostatics contribution to total energy. As much of the hydrogen bonding in ionic liquids is electrostatic, this could grant further insight into the energetics of hydrogen bonding. Since the electrostatics interaction is responsible for a significant part of the total interaction energy, these two energies are highly correlated. In Figure 14, the plot contrasts the HD bond lengths in both gas phase and implicit solvent, and the contribution of electrostatic interaction energy to the total interaction energy is given for both modes of optimization. Interestingly, for many of systems with the oxygen atom as an acceptor and the nitrogen atom as a donor, the relative electrostatic interaction energy is *negatively* correlated with increasing HD distance for gas phase optimized geometries. In particular, with increasing HD distance, the relative contribution from electrostatics also increases which is counter-intuitive to what is expected according to the Coulomb's law. As the solvent geometries have the HD bond distributed over a very narrow range of 1.03–1.81 Å, the length of this bond is likely not responsible for the change in the relative contribution from electrostatics. Referring to the relative contribution of electrostatics in gas phase optimized geometries, which is shown in the right-hand side of Figure 14, it is clear that gas phase optimizations may not account for all the electrostatic contributions present. The average relative contribution of electrostatics in gas-phase optimized structures is 85% compared to 91% for implicit solvent optimizations. As expected, the energy decomposition obtained using gas-phase geometries shows that an elongated donor-hydrogen bond is accompanied by a lower electrostatics component (exemplified by the two light red points in the Figure at longer HD distance).

**Figure 15:**
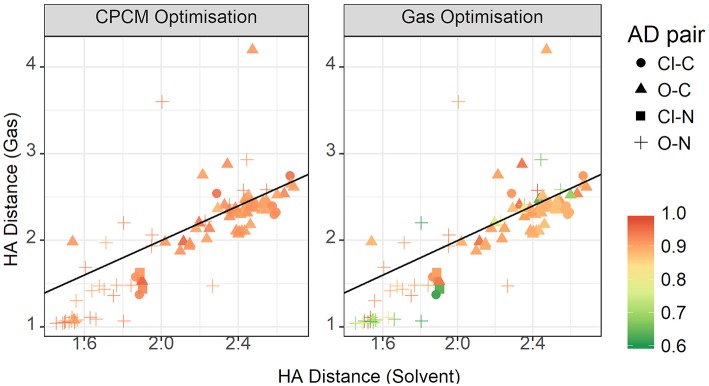
Gas vs. solvent optimized hydrogen bond distance in Å, colored by ratio of the electrostatic contribution to total CPCM interaction energy **(left)**, and the total gas interaction energy **(right)**.

The plot for the HA distances is shown in Figure 15, colored by the relative contribution of the electrostatic interaction to total interaction energy. This relative Coulomb force is strongest at short hydrogen bond distances, below approximately 1.8 Å. Additionally, there are some points around the 2.4 Å point that also have strong relative electrostatics. These points are not constrained to a particular acceptor-donor pair, but have a few representative each from oxygen-carbon, chloride-nitrogen, and oxygen-nitrogen. They are also not cation or anion specific. The strongest anions, from strongest to weakest, are trifluoroacetate, chloride, methylsulfate, trifluoromethanesulfonate, and methanesulfonate. Cations are even more diverse, with various members of the ammonium-based cations present, as well as N,N'-dimethyl pyrrolidinium and 1,3-dimethylimidazolium. The striking difference between the gas phase and implicit solvent geometries is the reduced contribution from electrostatics in the former.

Altogether, geometry and energies give a better picture of hydrogen bonding and its complexities. Solvent-optimized structures are shown again to be vital to produce the most accurate geometries, which would in hand produce reliable values when analyzing interaction energy.

## 4. Conclusions

Hydrogen bonding can be a difficult interaction to model, and even more so when present in ionic liquids. In this study of protic ionic liquids with clearly directional hydrogen bonding, geometry optimizations of single ion pairs were compared between gas phase and implicit solvent optimizations. Gas phase optimizations were found to produce shorter hydrogen bonding distance at least 0.7 Å on average. In many cases, geometries of ions pairs were destabilized in gas phase to such a degree that back proton transfer became preferred, resulting in the formation of neutral species. Implicit solvent optimizations, with ethanol as solvent, produced geometries that were in good agreement with available crystal structures of previously studied protic ionic liquids. The majority of the hydrogen bonding did not have perfectly linear hydrogen bonds, with the average angle being found at 150° experimentally, 143° with CPCM optimization, and 141° in gas phase optimizations.

Compared to aprotic ionic liquids, HBILs optimized in implicit solvent tend to have higher electrostatic energies, leading to a larger total interaction energy. In particular, the average value for total interaction energy is −409.1 kJ mol^−1^ for the HBIL dataset, whereas this number decreases to −329.2 kJ mol^−1^ on average for the IL174 dataset. The distribution of the interaction energy is much broader in HBILs, with the relative contribution remaining practically constant at 92%. In contrast to this, the IL174 dataset show a steeper dependence between the electrostatic interaction and total energy. The dispersion component was found to be similar in magnitude for both groups −36.3 kJ mol^−1^ on average for HBIL and −40.9 kJ mol^−1^ for IL174. In addition, for HBILs the electrostatic contribution was established to be 10 to 15 times greater than that for the dispersion energy. In the case of aprotic ionic liquids, the electrostatic contribution was 5–10 times greater than dispersion, thus making another clear energetic difference between the two classes of ionic liquids. In summary, geometric parameters and energetic components complement each other to better determine the presence of hydrogen bonding in ionic liquid systems. In particular, the total interaction energy and its electrostatic component can be reliably used to clearly classify the existence of hydrogen bonding as highlighted in Table 3.

Gas phase optimizations resulted in weaker interaction energies due to the fact that the gas phase tends to significantly reduce the electrostatic interaction. In this study we confirmed this commonly used rule, with the electrostatic contribution showing a decrease by 100 kJ mol^−1^ on average, compared to implicit solvent geometries. In addition, for many systems, especially imidazolium- and trimethyl ethyl ammonium-cation based ones, the absence of a stabilizing continuum model results in the ions becoming destabilized, resulting in the back proton transfer from the base to the acid. For some systems such as [EtMeNH_2_][TFA], the interaction energy was as strong as −760.3 kJ mol^−1^ observed due to the significantly decreased hydrogen bonding distance. Therefore, we conclude that gas phase optimizations should be avoided for studying hydrogen bonding in ionic liquids and protic ionic liquids as it produces unreliable geometries and energetics. Based on the data presented in this study, it is still inconclusive whether the hydrogen bond requires a special treatment in molecular dynamics simulations. The presence of a stronger electrostatic interaction might not necessary support this idea. Perhaps the only aspect that can be considered is the deviation from linearity of the hydrogen bond angle. Another aspect that has not been considered in this work is the charge distribution on the electronegative atoms partaking in hydrogen bonding. The presence of similar dispersion interactions might indicate that the charges in protic ionic liquids might have a similar distribution as that in aprotic ionic liquids.

## Author Contributions

SYST and KL ran calculations, which were analyzed by SYST, KL, and EII. SYST and KL equally edited the manuscript. KL, SYST, and EII approved the manuscript for publication.

### Conflict of Interest Statement

The authors declare that the research was conducted in the absence of any commercial or financial relationships that could be construed as a potential conflict of interest.
