# Supplementary material for: An ab initio Study of the Structure and Energetics of Hydrogen Bonding in Ionic Liquids
Source: Front Chem. 2019 Apr 10;7:208. doi: 10.3389/fchem.2019.00208 (PMC6468050; doi:10.3389/fchem.2019.00208)
Supplement: Supplementary file 1 [file Data_Sheet_1.PDF]

Supplementary Material: An *Ab Initio* Study of the Structure and Energetics of Hydrogen Bonding in Ionic Liquids

1 HBIL DATASET OPTIMISED GEOMETRIES

1.1 Gas-phase geometries

```
25
DMEA-CF3SO3-p1.log        Energy: -737673.5025638
C          1.78021        0.89418        1.19420
N          2.03030       -0.07517        0.10561
C          2.95957        0.42718       -0.94121
C          4.38469        0.65417       -0.46869
C          2.39608       -1.40581        0.63790
H          1.06561       -0.23785       -0.42415
H          3.29341       -1.33155        1.26648
H          1.53984       -1.76757        1.21993
H          1.49321        1.85499        0.74962
H          0.94679        0.50656        1.79209
H          2.51547        1.35794       -1.32145
H          2.92339       -0.30858       -1.75727
H          2.68315        1.01144        1.80845
H          2.57987       -2.08140       -0.20702
H          4.98004        1.03474       -1.30951
H          4.43837        1.39869        0.33785
H          4.85685       -0.27616       -0.12363
O         -0.13673       -0.55845       -1.14262
S         -1.22550       -0.86959       -0.12569
C         -2.05188        0.78043       -0.00657
O         -2.24726       -1.80352       -0.58749
O         -0.64175       -1.05425        1.22685
F         -3.00793        0.77039        0.91374
F         -1.14031        1.70406        0.34382
F         -2.57689        1.13748       -1.17171
18
DMEA-Cl-p1.log        Energy: -423247.6261382
C          0.45493        1.10811       -1.21941
N          0.29055        0.31034       -0.00001
C          1.03996       -0.96221       -0.00019
C          2.55316       -0.81682       -0.00001
C          0.45508        1.10782        1.21957
H         -0.89467       -0.02458        0.00002
H          1.45576        1.56241        1.27702
H         -0.31039        1.89504        1.21784
H          0.28415        0.46064       -2.08903
H         -0.31063        1.89525       -1.21745
H          0.69279       -1.51970       -0.88250
```

H	0.69264	-1.52001	0.88185
H	1.45556	1.56281	-1.27683
H	0.28429	0.46018	2.08906
H	3.01328	-1.81449	-0.00022
H	2.91353	-0.28555	-0.89275
H	2.91337	-0.28598	0.89305
Cl	-2.42602	-0.42234	0.00002

26

DMEA-mOSO3-p1.log Energy: -598077.5433813

C	1.37814	1.31222	0.64877
N	1.70178	-0.02859	0.11777
C	2.74908	-0.01108	-0.93543
C	4.13160	0.39248	-0.45193
C	1.95933	-0.99774	1.20212
H	0.75979	-0.38434	-0.40682
H	2.77994	-0.64856	1.84422
H	1.02839	-1.09568	1.77389
H	1.08976	1.95764	-0.19038
H	0.52117	1.19857	1.32318
H	2.38555	0.67308	-1.71512
H	2.76392	-1.02130	-1.36942
H	2.24277	1.73300	1.18027
H	2.22028	-1.96455	0.75274
H	4.82121	0.39134	-1.30675
H	4.13965	1.40401	-0.02225
H	4.52726	-0.31092	0.29370
H	-3.15040	2.60006	-0.22814
O	-1.88858	1.04492	-0.53274
O	-0.39944	-0.79046	-1.05102
C	-3.04753	1.56403	0.11528
H	-3.93534	0.98054	-0.16719
S	-1.56303	-0.52197	-0.11478
O	-2.76187	-1.30766	-0.38949
H	-2.92077	1.54019	1.20887
O	-1.10021	-0.45442	1.29430

25

DMEA-mSO3-p1.log Energy: -550891.8540946

C	1.62525	1.56001	0.24477
N	1.46907	0.12032	0.01103
C	2.51203	-0.45477	-0.85655
C	3.94115	-0.34112	-0.33908
C	1.30447	-0.60606	1.27723
H	0.23476	-0.00784	-0.67116
H	2.21152	-0.54908	1.90183
H	0.46015	-0.16294	1.82191
H	1.79563	2.06403	-0.71697
H	0.69005	1.93257	0.68487
H	2.42410	0.04620	-1.83339
H	2.24485	-1.51104	-1.01289
H	2.46735	1.77876	0.92290
H	1.06059	-1.65524	1.06280
H	4.62776	-0.81155	-1.05656
H	4.24951	0.70789	-0.22353
H	4.07035	-0.84981	0.62672

```
O        -0.76924      -0.08202      -1.13989
S        -1.80931      -0.08576       0.05090
C        -3.31739       0.20066      -0.84838
O        -1.86818      -1.41239       0.68080
O        -1.54569       1.07426       0.92854
H        -4.12427       0.21230      -0.10702
H        -3.22946       1.16679      -1.35558
H        -3.44553      -0.62240      -1.55906
21
DMEA-NO3-p1.log        Energy: -310314.0092344
C         0.96606       0.95819      -1.20803
N         0.91954       0.13103       0.00013
C         1.91868      -0.95258       0.00118
C         3.37800      -0.51212       0.00001
C         0.96624       0.96070       1.20652
H        -0.39788      -0.52254       0.00028
H         1.87018       1.59279       1.24065
H         0.07470       1.60050       1.21654
H         0.94765       0.30727      -2.09323
H         0.07444       1.59787      -1.21927
H         1.70968      -1.57598      -0.88214
H         1.71036      -1.57368       0.88627
H         1.86995       1.59027      -1.24356
H         0.94787       0.31164       2.09309
H         4.02726      -1.39853       0.00099
H         3.62600       0.07958      -0.89267
H         3.62668       0.08205       0.89086
O        -1.33638      -1.06134       0.00029
N        -2.30235      -0.14452       0.00008
O        -3.42717      -0.55623       0.00025
O        -1.95909       1.02733      -0.00020
24
DMEA-TFA-p1.log        Energy: -464594.3241526
C        -2.26152      -1.67963      -0.61161
N        -2.20955      -0.41593       0.12820
C        -2.43844       0.71331      -0.79261
C        -2.23856       2.06677      -0.12776
C        -3.15725      -0.42960       1.24189
H        -0.78861      -0.25671       0.59847
H        -4.20105      -0.48838       0.87931
H        -2.95337      -1.30196       1.87692
H        -1.48877      -1.66141      -1.39123
H        -2.05886      -2.51221       0.07588
H        -3.45488       0.63213      -1.22617
H        -1.70976       0.59124      -1.60610
H        -3.25437      -1.83101      -1.07359
H        -3.04073       0.47504       1.85141
H        -2.25015       2.85604      -0.89157
H        -3.02917       2.29853       0.59954
H        -1.26710       2.10378       0.38792
F         3.18013       0.02707      -1.01485
F         2.88621      -0.96622       0.88142
C         2.45255       0.02559       0.09603
C         0.95669      -0.15407      -0.22107
```

```
O          0.57383       -0.29898       -1.35884
O          0.23934       -0.12671        0.86614
F          2.66448        1.17889        0.73945
22
EtMeNH2-CF3SO3-p1.log        Energy: -713017.5923186
C         -2.53416       -1.64644        0.23778
N         -1.89559       -0.35031       -0.06100
C         -2.75509        0.70723       -0.63588
C         -3.85643        1.15451        0.30805
H         -1.41676       -0.02294        0.79012
H         -1.02672       -0.56051       -0.70847
H         -2.96765       -2.04702       -0.68700
H         -1.74364       -2.31002        0.60655
H         -3.31384       -1.53004        0.99973
H         -4.40000        1.99792       -0.13763
H         -4.58408        0.35485        0.50290
H         -3.43833        1.49279        1.26797
H         -3.16478        0.31238       -1.57648
H         -2.08311        1.53950       -0.88400
O          0.30630       -0.91654       -1.25044
S          1.20806       -0.86920       -0.03042
C          1.51702        0.95369        0.06832
O          0.42171       -1.10550        1.21049
O          2.50678       -1.51309       -0.17981
F          2.20632        1.27886        1.15262
F          2.14489        1.41467       -1.00293
F          0.31243        1.57308        0.14237
15
EtMeNH2-Cl-p1.log        Energy: -398591.5735156
C          0.47887        1.61892       -0.19791
N          0.35859        0.34895        0.52582
C          1.09154       -0.78454       -0.06564
C          2.60036       -0.60716       -0.11456
H          0.60936        0.46532        1.50995
H         -0.88682        0.02661        0.39503
H          0.11472        1.45408       -1.22122
H         -0.17750        2.35685        0.27943
H          1.51045        1.99717       -0.22165
H          3.06825       -1.51811       -0.51228
H          2.89609        0.22862       -0.76406
H          3.01346       -0.42915        0.89037
H          0.66487       -0.92901       -1.06952
H          0.81586       -1.67225        0.52088
Cl        -2.30374       -0.34036       -0.07171
23
EtMeNH2-mOSO3-p1.log        Energy: -573423.0583331
C         -3.00506        1.29647        0.65544
N         -1.82786        0.52937        0.23183
C         -1.92884       -0.94894        0.36337
C         -2.73669       -1.56655       -0.76188
H         -1.54947        0.72444       -0.74416
H         -0.87845        0.82090        0.72417
H         -3.18710        1.11132        1.72108
H         -2.81087        2.36409        0.50224
```

```
H          -3.88850        0.99902        0.07659
H          -3.76980       -1.19061       -0.79254
H          -2.24669       -1.36173       -1.72518
H          -2.78105       -2.65581       -0.63125
H          -2.36367       -1.15440        1.35246
H          -0.89646       -1.32436        0.35047
O           0.26173        0.34031       -1.42715
O           1.55782       -1.13452        0.00773
S           1.24750        0.45596       -0.32382
O           2.52707        1.10237       -0.59587
H           2.67607       -2.40264        1.12328
O           0.57332        0.94570        0.94920
C           2.56910       -1.31898        0.99725
H           3.51696       -0.87913        0.65691
H           2.26332       -0.85508        1.94783
22
EtMeNH2-mSO3-p1.log         Energy: -526236.2882700
C          -1.52147        1.48137        0.10787
N          -1.46698        0.00730        0.09943
C          -2.70026       -0.69577        0.49293
C          -3.86755       -0.44051       -0.44638
H          -1.12194       -0.30056       -0.82329
H          -0.52629       -0.27683        0.72393
H          -1.84557        1.81406        1.10278
H          -0.50747        1.85782       -0.08651
H          -2.21404        1.85942       -0.65452
H          -4.72725       -1.05554       -0.14857
H          -4.18593        0.61097       -0.42723
H          -3.60310       -0.70544       -1.48115
H          -2.94093       -0.38105        1.51905
H          -2.45432       -1.76601        0.52673
O           0.75790       -0.43853        1.14515
S           1.59363        0.02260       -0.06790
C           3.06607       -0.97569        0.03321
H           3.69655       -0.70465       -0.82076
H           3.56214       -0.73953        0.98070
H           2.76494       -2.02743       -0.01176
O           0.87608       -0.35278       -1.32467
O           1.99267        1.43952        0.03766
18
EtMeNH2-NO3-p1.log         Energy: -285658.7903692
C          -1.07030        1.31505        0.71503
N          -0.98001        0.07260       -0.05717
C          -2.13962       -0.81920        0.06757
C          -3.45606       -0.23622       -0.43140
H          -0.82034        0.31042       -1.03950
H           0.34920       -0.50915        0.31440
H          -1.19243        1.06230        1.77836
H          -0.12876        1.86418        0.58612
H          -1.90747        1.96047        0.40639
H          -4.25471       -0.98755       -0.35850
H          -3.76569        0.63786        0.15850
H          -3.37510        0.07023       -1.48591
H          -2.22116       -1.09723        1.13038
```

```
H        -1.89992      -1.74022      -0.48391
O         1.33045      -0.83709       0.58699
N         2.17445      -0.08293      -0.12037
O         3.34060      -0.30675       0.03244
O         1.68535       0.76175      -0.85327
21
EtMeNH2-TFA-p1.log        Energy: -439938.3835567
C        -3.37389      -1.32351       0.47893
N        -2.27177      -0.65424      -0.21122
C        -2.60830       0.68627      -0.72186
C        -2.75670       1.69770       0.40500
H        -0.92926      -0.47842       0.50753
H        -1.96761      -1.21886      -1.00766
H        -4.29347      -1.34933      -0.13151
H        -3.07996      -2.35170       0.72549
H        -3.59498      -0.80058       1.41869
H        -3.61436       1.46911       1.05382
H        -1.84668       1.71907       1.02246
H        -2.91483       2.70182      -0.01151
H        -3.53085       0.64632      -1.33019
H        -1.78171       0.97816      -1.38520
O         0.42914      -0.35522      -1.40291
F         2.40028       1.17461       0.77532
F         3.00863       0.13136      -1.01669
C         2.27284       0.03709       0.08418
C         0.79520      -0.22642      -0.25803
O         0.06359      -0.28493       0.82094
F         2.76213      -0.95503       0.83505
19
EtNH3-CF3SO3-p1.log        Energy: -688363.0186561
C         2.35782       1.54171       0.11154
C         3.34564       0.40517      -0.07958
N         2.61605      -0.88868      -0.05266
H         1.73161      -0.86819      -0.72169
H         3.21337      -1.69604      -0.23829
H         2.11611      -1.01350       0.84781
H         4.10948       0.38730       0.71013
H         3.85661       0.47438      -1.04933
H         2.89457       2.49875       0.13820
H         1.63009       1.55995      -0.71000
H         1.79628       1.41636       1.04788
O         0.33004      -0.74880      -1.18259
S        -0.55764      -0.87640       0.04572
C        -1.54010       0.68765      -0.02955
O        -1.54069      -1.95467      -0.00314
O         0.25580      -0.73038       1.28939
F        -2.35371       0.76144       1.01659
F        -0.72873       1.74988      -0.00973
F        -2.25973       0.72732      -1.14367
12
EtNH3-Cl-p1.log        Energy: -373937.8603154
C         1.63073      -1.08896      -0.21349
C         1.51225       0.25341       0.48774
N         0.62292       1.14136      -0.28290
```

```
H         -0.70354        0.53205       -0.19277
H          0.59066        2.08007        0.11817
H          0.95046        1.23615       -1.24675
H          2.50431        0.71001        0.63592
H          1.04762        0.12225        1.47607
H          2.25328       -1.77407        0.37702
H          0.63527       -1.53625       -0.34209
H          2.09848       -0.97538       -1.20413
Cl        -1.91734       -0.19830        0.04196
20
EtNH3-mOSO3-p1.log        Energy: -548769.7884194
C          2.52454        1.31220       -0.20686
C          2.41566        0.01111        0.56910
N          2.07544       -1.09625       -0.34811
H          0.80490       -0.75159       -0.97531
H          2.85590       -1.30525       -0.97333
H          1.86481       -1.94155        0.18742
H          1.59750        0.07245        1.29999
H          3.34707       -0.20070        1.11879
H          2.74114        2.14752        0.47253
H          3.33025        1.26583       -0.95624
H          1.57738        1.51970       -0.72542
O         -0.44721       -1.41143        1.00896
O         -0.81060        0.97380        0.67629
S         -1.07275       -0.51115        0.04216
O         -0.20372       -0.45672       -1.26606
H         -1.18619        2.95424        0.51388
O         -2.47751       -0.61474       -0.31490
C         -1.46167        2.04007       -0.02305
H         -1.10617        2.08943       -1.06359
H         -2.54959        1.89463       -0.00584
19
EtNH3-mSO3-p1.log        Energy: -501583.7687277
C         -2.27696        1.27120       -0.09108
C         -2.91943       -0.09765        0.07704
N         -1.88307       -1.14364        0.04070
H         -0.63243       -0.74039        0.78899
H         -2.28162       -2.06485        0.22463
H         -1.45307       -1.17258       -0.89031
H         -3.68250       -0.26167       -0.70205
H         -3.42798       -0.16247        1.05030
H         -3.04320        2.05813       -0.11146
H         -1.58752        1.47019        0.74257
H         -1.69763        1.31150       -1.02549
O          0.29450       -0.36263        1.15989
S          1.26631       -0.13181       -0.07905
C          1.78857        1.54486        0.21441
O          2.43636       -1.00183        0.02124
O          0.45556       -0.16014       -1.31780
H          2.50416        1.79286       -0.57795
H          0.90828        2.19521        0.17360
H          2.26964        1.57494        1.19817
15
EtNH3-NO3-p1.log        Energy: -261006.2714569
```

```
C         -1.74088       -1.30774       -0.19402
C         -2.38618        0.06784       -0.14500
N         -1.39849        1.07442        0.28319
H         -0.04503        0.87416       -0.43051
H         -1.80816        2.00938        0.28602
H         -1.09531        0.87574        1.24060
H         -3.26538        0.05768        0.52102
H         -2.73941        0.35620       -1.14614
H         -2.47466       -2.06435       -0.50348
H         -0.90715       -1.31631       -0.91092
H         -1.34302       -1.59160        0.79137
O          0.88250        0.64385       -0.87768
N          1.59285       -0.00940        0.05203
O          2.69040       -0.35442       -0.27579
O          1.06210       -0.19151        1.13343
18
EtNH3-TFA-p1.log        Energy: -415285.1225612
C          4.27223        0.65255        0.21216
C          2.93068        0.01642        0.54203
N          2.38484       -0.82577       -0.53483
H          2.24086       -0.24414       -1.36529
H          3.05300       -1.55476       -0.79213
H          0.90969       -1.17378       -0.14772
H          2.17679        0.79152        0.74233
H          3.00868       -0.60727        1.44512
H          4.62631        1.27196        1.04844
H          5.03699       -0.11400        0.01213
H          4.19138        1.29762       -0.67594
O          0.02966        0.92475       -0.71918
F         -2.56196        1.28349       -0.29174
F         -2.29808       -0.04801        1.38973
C         -2.08054        0.10290        0.07876
C         -0.57640       -0.00050       -0.23232
O         -0.10614       -1.17171        0.10074
F         -2.76235       -0.85096       -0.56237
21
mim-CF3SO3-p1.log        Energy: -770139.4370443
C          3.49509        0.93980        0.46236
N          2.31270        0.77863       -0.22863
C          2.18233       -0.49279       -0.58736
N          3.26566       -1.14589       -0.15942
C          4.10332       -0.27659        0.50892
C          1.26847        1.78689       -0.41298
H          3.40715       -2.14158       -0.28037
H          1.29782       -0.88929       -1.10601
H          5.04137       -0.59172        0.94900
H          3.79904        1.90008        0.86283
H          1.72450        2.70271       -0.80681
H          0.52922        1.37604       -1.10892
H          0.77521        1.95232        0.55061
O         -0.55389       -0.74238       -1.37555
S         -1.07357       -0.85979        0.02979
O         -1.82971       -2.07767        0.33568
O         -0.05780       -0.42411        1.03047
```

```
C        -2.32853       0.50127       0.09097
F        -2.87058       0.59026       1.30294
F        -1.74638       1.68323      -0.18583
F        -3.29844       0.30658      -0.79676
21
mim-CF3SO3-p2.log        Energy: -770149.2161080
C         3.73419       0.63126       0.74791
N         3.37420      -0.37556      -0.11961
C         2.06846      -0.21435      -0.41362
N         1.57708       0.83796       0.21636
C         2.60498       1.37875       0.94741
C         4.24083      -1.42205      -0.63140
H         0.24376       1.25303       0.07321
H         1.49607      -0.85669      -1.07834
H         2.46790       2.26141       1.56331
H         4.74298       0.72163       1.13554
H         4.61645      -2.04101       0.19338
H         3.66148      -2.05002      -1.31707
H         5.08527      -0.97951      -1.17477
O        -0.81699       1.54757      -0.06921
S        -1.58871       0.39846      -0.78487
O        -2.79357       0.89321      -1.43047
O        -0.65543      -0.49154      -1.49117
C        -2.12523      -0.58011       0.68949
F        -2.69221      -1.71405       0.29820
F        -1.05182      -0.86749       1.42778
F        -2.98542       0.10643       1.42664
14
mim-Cl-p1.log        Energy: -455722.4724010
C         1.77233       1.13746       0.00022
N         1.77217      -0.23819      -0.00094
C         0.47792      -0.63497      -0.00126
N        -0.33817       0.39812      -0.00066
C         0.45480       1.51608       0.00023
C         2.93514      -1.10834       0.00110
H        -1.90165       0.11554      -0.00006
H         0.17299      -1.67890      -0.00197
H         0.03312       2.51592       0.00053
H         2.69447       1.70862       0.00055
H         3.83676      -0.48679      -0.01180
H         2.94565      -1.73195       0.90403
H         2.93345      -1.75078      -0.88850
Cl       -3.21141      -0.31015       0.00039
14
mim-Cl-p2.log        Energy: -455707.8145271
C         1.60450       0.15726      -0.65089
N         0.72340       0.76910       0.23678
C         0.00802      -0.19720       0.85596
N         0.63741      -1.37095       0.60301
C         1.54883      -1.17483      -0.42863
C         0.23198       2.12911       0.12198
H         0.19203      -2.25503       0.81174
H        -0.58798      -0.01560       1.74299
H         2.09355      -1.99264      -0.88334
```

```
H          2.20886        0.73594       -1.33942
H          0.99891        2.74297       -0.36357
H         -0.69897        2.11080       -0.46497
H          0.02954        2.53340        1.12169
Cl        -2.00716       -0.30194       -0.34672
22
mim-mOSO3-p1.log        Energy: -630543.3268184
C          2.86959       -0.06063       -0.67313
N          1.98979        0.68571        0.08169
C          1.30106       -0.12539        0.87331
N          1.73440       -1.36815        0.66583
C          2.71228       -1.36194       -0.30630
C          1.65809        2.09671       -0.11599
H          1.29599       -2.17790        1.08914
H          0.46630        0.19032        1.50388
H          3.20323       -2.26443       -0.64859
H          3.52115        0.39371       -1.41035
H          2.52531        2.71810        0.13679
H          1.34928        2.22120       -1.15966
H          0.79073        2.31917        0.51714
H         -1.11331       -2.05865       -1.25751
O         -1.15931        1.26157        1.14502
H         -2.81046       -1.93909       -0.68751
C         -1.76760       -2.08934       -0.36998
S         -1.58219        0.47543       -0.05002
O         -1.38803       -1.09434        0.56734
H         -1.67582       -3.06067        0.13606
O         -0.58306        0.51690       -1.15309
O         -2.98802        0.57485       -0.44666
21
mim-mSO3-p1.log        Energy: -583370.6997789
C         -3.28685        0.92509        0.07725
N         -2.87023       -0.38360       -0.01937
C         -1.52440       -0.37321       -0.13650
N         -1.05894        0.86194       -0.11881
C         -2.14905        1.68512        0.01445
C         -3.71706       -1.56089       -0.00938
H          0.44657        1.12290       -0.20208
H         -0.90140       -1.26167       -0.22230
H         -2.04954        2.76477        0.06033
H         -4.33436        1.18674        0.18137
H         -4.32057       -1.58585        0.90692
H         -4.37992       -1.56355       -0.88454
H         -3.07792       -2.45019       -0.04043
O          1.29493       -1.26462       -0.39152
S          2.14287       -0.17674        0.13972
O          1.49523        1.22010       -0.25113
O          2.51184       -0.20948        1.55595
C          3.62764       -0.08787       -0.83588
H          4.18092        0.80386       -0.52239
H          4.19809       -0.99840       -0.62072
H          3.33874       -0.03669       -1.89053
21
mim-mSO3-p2.log        Energy: -583359.1302375
```

```
C        -2.51652      -0.35072       0.77418
N        -1.99463       0.56956      -0.11409
C        -1.19588      -0.06871      -0.96040
N        -1.20651      -1.36510      -0.66607
C        -2.00559      -1.56597       0.43364
C        -2.00352       2.02527       0.00813
H        -0.37261      -1.90414      -0.94141
H        -0.52977       0.40499      -1.67509
H        -2.12898      -2.53814       0.89360
H        -3.18307      -0.05758       1.57621
H        -2.49057       2.28709       0.95285
H        -0.95074       2.34472       0.01501
H        -2.54984       2.47633      -0.82872
O         0.70621      -0.24017       1.27209
S         1.51327       0.03999       0.04866
O         1.45655      -1.08703      -0.95614
O         1.17910       1.36648      -0.57523
C         3.22669       0.14480       0.55001
H         3.82372       0.34697      -0.34568
H         3.31091       0.96147       1.27535
H         3.50057      -0.81512       1.00129
17
mim-NO3-p1.log       Energy: -342793.1480849
C        -2.61332       1.00010       0.00059
N        -2.30969      -0.34187       0.00001
C        -0.96160      -0.44972      -0.00055
N        -0.39158       0.73977      -0.00030
C        -1.41149       1.65765       0.00009
C        -3.25380      -1.44587      -0.00017
H         1.15159       0.88105      -0.00048
H        -0.42347      -1.39395      -0.00116
H        -1.22122       2.72586       0.00026
H        -3.63835       1.35474       0.00111
H        -4.27018      -1.03734       0.00240
H        -3.12000      -2.06311      -0.89776
H        -3.11669      -2.06604       0.89488
O         2.19242       0.98782      -0.00041
N         2.70281      -0.24481       0.00015
O         3.89851      -0.31777      -0.00004
O         1.91766      -1.17577       0.00071
17
mim-NO3-p2.log       Energy: -342773.0579783
C         1.97454      -0.40338       0.57854
N         1.37428       0.53255      -0.24114
C         0.47143      -0.08185      -0.99586
N         0.48273      -1.37837      -0.69813
C         1.40278      -1.60606       0.29794
C         1.39761       1.98451      -0.06488
H        -0.34715      -1.93954      -0.90606
H        -0.26495       0.40865      -1.62724
H         1.55822      -2.58489       0.73340
H         2.73620      -0.12833       1.29829
H         2.25411       2.24579       0.56435
H         1.49401       2.46973      -1.04236
```

```
H          0.44088        2.25607        0.40597
O         -2.17078       -0.73179       -0.50574
N         -1.71176        0.12534        0.28646
O         -1.68348        1.32628       -0.06590
O         -1.19150       -0.22492        1.35276
20
mim-TFA-p1.log        Energy: -497051.4211431
C          3.97354       -0.35540        0.08729
N          2.62900       -0.64592       -0.04481
C          1.91873        0.48448       -0.08254
N          2.80335        1.48700        0.02402
C          4.08782        0.99935        0.13225
C          2.05496       -1.99020       -0.14150
H          2.52808        2.46242        0.02705
H          0.76233        0.63104       -0.18173
H          4.95540        1.64052        0.23046
H          4.72582       -1.13425        0.14167
H          2.42857       -2.59214        0.69573
H          0.96013       -1.88767       -0.08224
H          2.36098       -2.44252       -1.09303
F         -3.23257        1.03693        0.91417
O         -0.72223        0.98903       -0.26034
C         -1.33747       -0.08214       -0.01412
F         -3.51186       -1.03197        0.36289
O         -0.88956       -1.21593        0.18290
C         -2.87475        0.09278        0.02908
F         -3.34340        0.47915       -1.16992
20
mim-TFA-p2.log        Energy: -497048.8648628
C          3.40108       -0.35851       -0.21332
N          2.42852        0.60940       -0.03950
C          1.27172        0.03990        0.30422
N          1.49319       -1.27647        0.34859
C          2.80481       -1.55805        0.03172
C          2.56819        2.05189       -0.21407
H          0.74091       -1.92953        0.54896
H          0.27138        0.63742        0.44688
H          3.19934       -2.56650        0.00229
H          4.41988       -0.11609       -0.49380
H          1.56796        2.48975       -0.10235
H          3.24692        2.45746        0.54598
H          2.95845        2.26264       -1.21693
F         -3.04469       -1.12867       -0.29303
O         -3.11850        1.51605        0.01960
C         -2.00890        1.03571        0.12924
F         -1.26347       -1.13265        0.93908
O         -0.88397        1.56082        0.39817
C         -1.89209       -0.50647       -0.11087
F         -1.10362       -0.78395       -1.17920
20
mim-TFA-p3.log        Energy: -497034.2943797
C          1.60958       -0.45481       -0.15426
N          2.97766       -0.30444       -0.01967
C          3.25436        0.99087        0.12189
```

```
N         2.09500        1.65172        0.07835
C         1.03680        0.78525       -0.09419
C         3.95345       -1.38606       -0.02787
H         1.99808        2.65797        0.16462
H         4.24293        1.41848        0.24873
H        -0.06414        1.13986       -0.13204
H         1.14164       -1.42539       -0.27674
H         4.95603       -0.96544        0.10300
H         3.73511       -2.07877        0.79336
H         3.89954       -1.91766       -0.98521
F        -1.13690       -0.82992        1.12001
O        -3.66885        0.97853       -0.02776
C        -2.45970        0.83166       -0.04330
F        -2.89685       -1.56325        0.10210
O        -1.51098        1.66524       -0.11427
C        -1.94216       -0.64223        0.04167
F        -1.15439       -0.95776       -1.02809
26
mpyr-CF3SO3-p1.log        Energy: -761583.6597728
C         2.90455        0.00546       -1.13902
N         2.28404        0.79970       -0.04457
C         2.49806       -0.01235        1.18989
C         2.35676       -1.46853        0.72122
C         2.44949       -1.41711       -0.82412
C         2.73087        2.19489        0.05211
H         1.20963        0.78926       -0.29253
H         2.20243        2.67850        0.88214
H         3.81518        2.22529        0.22949
H         2.48959        2.71285       -0.88397
H         2.54863        0.39152       -2.10255
H         3.99723        0.12911       -1.06765
H         1.45488       -1.59231       -1.25587
H         3.14484       -2.15720       -1.23718
H         1.38443       -1.87398        1.02864
H         3.14664       -2.08738        1.16475
H         1.74662        0.28027        1.93212
H         3.51277        0.21535        1.55122
O        -0.13840        0.58995       -0.84856
S        -0.87680       -0.34538        0.09682
C        -2.61601        0.25398       -0.07615
O        -0.89016       -1.74457       -0.35645
O        -0.51082       -0.09109        1.50743
F        -3.41823       -0.45904        0.70716
F        -2.69789        1.53334        0.27819
F        -3.02161        0.13067       -1.33521
19
mpyr-Cl-p1.log        Energy: -447158.8897519
C         0.93853        0.25016       -1.17002
N         0.20090        0.72204        0.03515
C         0.82899        0.11210        1.22655
C         1.21688       -1.26424        0.70576
C         1.77649       -0.94719       -0.68814
C        -0.04423        2.16179        0.10419
H        -0.86713        0.19506       -0.02720
```

H	-0.67348	2.36909	0.97913
H	0.90409	2.71569	0.18027
H	-0.58890	2.46917	-0.79761
H	0.18161	-0.02813	-1.91741
H	1.55688	1.06730	-1.56855
H	1.70414	-1.79883	-1.37481
H	2.83582	-0.66036	-0.61654
H	0.30324	-1.87084	0.62303
H	1.93589	-1.77715	1.35588
H	0.08717	0.09085	2.03656
H	1.70518	0.70870	1.53261
Cl	-2.28181	-0.61239	-0.10126

27

mpyr-mOSO3-p1.log Energy: -621988.0493839

C	1.79482	0.09774	-1.25785
N	1.67599	0.84630	0.04173
C	2.13301	-0.07191	1.14754
C	2.76288	-1.26670	0.43720
C	1.93902	-1.36294	-0.84811
C	2.34476	2.15572	0.02286
H	0.58173	1.02040	0.20327
H	2.11903	2.68812	0.95481
H	3.43131	2.02125	-0.07966
H	1.96115	2.73606	-0.82573
H	0.89526	0.29656	-1.85488
H	2.69363	0.46400	-1.77717
H	0.94765	-1.78865	-0.64007
H	2.42309	-1.95551	-1.63444
H	2.71073	-2.17226	1.05368
H	3.82116	-1.06604	0.20461
H	1.23054	-0.37299	1.69602
H	2.81484	0.47435	1.81109
O	-0.81981	-1.30561	1.02368
O	-2.94524	-0.18158	0.53423
O	-1.32273	-0.61644	-1.33489
S	-1.37762	-0.29985	0.10578
H	-4.72249	0.68662	0.11764
H	-3.66663	0.42971	-1.31963
C	-3.69638	0.73326	-0.26367
O	-0.82536	1.09737	0.38074
H	-3.29459	1.75093	-0.15201

26

mpyr-mSO3-p1.log Energy: -574803.9430294

C	2.20319	-0.19362	-1.07989
N	1.60961	0.69335	-0.05351
C	1.69162	-0.07964	1.21377
C	1.41456	-1.53113	0.79310
C	1.58851	-1.55089	-0.74588
C	2.18581	2.03652	0.01822
H	0.46440	0.79520	-0.37749
H	1.66821	2.60219	0.80276
H	3.26101	1.98059	0.24978
H	2.04028	2.54191	-0.94488
H	1.94396	0.18494	-2.07776

```
H          3.30030      -0.18573      -0.95855
H          0.59970      -1.63694      -1.21676
H          2.21971      -2.37558      -1.09838
H          0.38675      -1.81073       1.05700
H          2.10642      -2.21543       1.30045
H          0.94512       0.31322       1.91372
H          2.70912       0.04991       1.61862
O         -0.74715       0.85471      -0.85491
S         -1.67068      -0.02835       0.02242
C         -3.27944       0.68531      -0.26458
O         -1.70243      -1.42372      -0.46551
O         -1.33383       0.15898       1.45583
H         -3.99526       0.10056       0.32389
H         -3.25054       1.72829       0.06747
H         -3.49377       0.60854      -1.33575
22
mpyr-NO3-p1.log        Energy: -334224.5282115
C          1.34681      -0.26689      -1.16110
N          1.04986       0.69524      -0.07798
C          1.62097       0.10193       1.14052
C          1.19629      -1.35639       1.01960
C          1.35403      -1.65199      -0.48548
C          1.51452       2.04891      -0.35680
H         -0.39942       0.79607       0.18459
H          1.22616       2.71330       0.46950
H          2.61271       2.07983      -0.47866
H          1.04292       2.41441      -1.27915
H          0.58337      -0.17394      -1.94403
H          2.33503      -0.02842      -1.59508
H          0.53282      -2.27787      -0.85158
H          2.30187      -2.16824      -0.68963
H          0.14001      -1.45037       1.30812
H          1.79096      -2.02131       1.65793
H          1.22387       0.61463       2.02861
H          2.72390       0.20555       1.13396
O         -1.42447       0.98411       0.47312
N         -2.15588      -0.00698      -0.03464
O         -3.33712       0.05203       0.15427
O         -1.55938      -0.88300      -0.63946
25
mpyr-TFA-p1.log        Energy: -488503.2855727
C          2.66401       0.11543      -1.15392
N          2.07497       0.68509       0.06599
C          2.36205      -0.30897       1.11851
C          2.23049      -1.67220       0.41583
C          2.19942      -1.33681      -1.09428
C          2.54930       2.02509       0.38578
H          0.59082       0.74642      -0.20711
H          2.06644       2.37359       1.30881
H          3.64590       2.03904       0.52854
H          2.28580       2.71338      -0.42899
H          2.31039       0.66498      -2.03820
H          3.76879       0.18927      -1.11044
H          1.17210      -1.40362      -1.48017
```

```
H          2.82995       -2.00016       -1.69894
H          1.30869       -2.17685        0.72721
H          3.08052       -2.31882        0.66945
H          1.64959       -0.18539        1.94376
H          3.38998       -0.14806        1.49449
F         -3.32442       -0.74083        0.75303
O         -0.71103       -0.69666        1.19233
C         -1.12292        0.00206        0.29704
F         -3.10623        1.26472       -0.02052
O         -0.44053        0.79955       -0.47539
C         -2.61743        0.02145       -0.07287
F         -2.79167       -0.43376       -1.31943
28
TMEA-CF3SO3-p1.log         Energy: -762313.4673478
C          2.10526       -0.39061        1.25741
N          2.58698        0.27366        0.00159
C          4.09039        0.33693       -0.00587
C          4.78458       -1.01100        0.00179
C          2.09477       -0.45575       -1.21485
C          2.06116        1.67633       -0.03283
H          2.25940       -1.52891       -1.07633
H          1.02486       -0.25684       -1.34604
H          2.62508        0.08264        2.10050
H          1.02242       -0.24892        1.34715
H          4.37136        0.92887        0.87619
H          4.36226        0.91321       -0.90120
H          2.33995       -1.45851        1.20566
H          2.66280       -0.08098       -2.07647
H          5.86850       -0.83494       -0.00783
H          4.55181       -1.59290        0.90291
H          4.53764       -1.60898       -0.88489
O         -0.87432        0.82551        1.21785
S         -1.17881        0.03323       -0.00601
C         -3.02100       -0.13077        0.00334
O         -0.87615        0.75320       -1.27375
O         -0.67008       -1.36163        0.03527
F         -3.43162       -0.82430       -1.05662
F         -3.42271       -0.76307        1.10447
F         -3.59387        1.07053       -0.02795
H          2.46391        2.21122        0.83601
H          0.96554        1.64774        0.00509
H          2.40391        2.14405       -0.96374
21
TMEA-Cl-p1.log         Energy: -447885.3957385
C          0.40059        0.36909       -1.40314
N          0.91936        0.37817        0.00277
C          0.45104       -0.84403        0.78003
C          0.56055       -2.14655        0.01492
C          2.40081        0.43743       -0.01531
C          0.37124        1.58521        0.69851
H          2.79303       -0.48242       -0.46432
H          2.71201        1.30385       -0.61150
H         -0.69730        0.25192       -1.34203
H          0.67631        1.32852       -1.85924
```

H	-0.61074	-0.64810	1.00501
H	1.06056	-0.84478	1.69546
H	0.87939	-0.45416	-1.94463
H	2.76571	0.53955	1.01391
H	0.34790	-2.96122	0.72033
H	-0.20491	-2.19067	-0.77132
H	1.55660	-2.33326	-0.41489
Cl	-2.56251	0.11086	-0.00389
H	-0.72835	1.52043	0.62538
H	0.76873	2.47864	0.20050
H	0.70274	1.55294	1.74412

29

TMEA-mOSO3-p1.log Energy: -622717.6795989

C	1.57856	-0.38768	1.24264
N	2.01479	0.33626	0.00015
C	3.51001	0.49725	0.00005
C	4.28361	-0.80727	-0.00088
C	1.57823	-0.38586	-1.24324
C	1.39245	1.69869	0.00122
H	1.79560	-1.45237	-1.13115
H	0.50099	-0.23909	-1.38615
H	2.14140	0.04041	2.08295
H	0.50145	-0.24076	1.38626
H	3.75047	1.09626	0.88943
H	3.75023	1.09740	-0.88864
H	1.79533	-1.45411	1.12867
H	2.14045	0.04386	-2.08314
H	5.35579	-0.56993	-0.00070
H	4.07481	-1.40998	0.89243
H	4.07485	-1.40873	-0.89504
H	-3.96188	1.09396	0.89759
O	-3.26924	-0.64955	0.00062
O	-1.44354	0.55674	-1.23477
C	-4.13247	0.48172	-0.00091
H	-3.96382	1.09005	-0.90240
S	-1.67379	-0.24929	0.00017
O	-1.44291	0.55689	1.23491
H	-5.15451	0.08451	0.00115
O	-0.98733	-1.55872	0.00014
H	1.72863	2.22565	0.90269
H	0.30090	1.58659	0.00147
H	1.72823	2.22692	-0.89965

28

TMEA-mSO3-p1.log Energy: -575531.0876374

C	-1.25042	-0.37214	-1.26107
N	-1.71552	0.29609	0.00025
C	-3.21541	0.39302	0.00922
C	-3.93591	-0.94121	-0.00297
C	-1.23914	-0.44536	1.21731
C	-1.15394	1.68450	0.03868
H	-1.39634	-1.51781	1.06409
H	-0.17001	-0.24211	1.36393
H	-1.79581	0.09207	-2.09481
H	-0.16965	-0.21741	-1.37040

```
H        -3.48586        0.99354       -0.87055
H        -3.47603        0.97100        0.90697
H        -1.47281       -1.44141       -1.19752
H        -1.82514       -0.07977        2.07159
H        -5.01687       -0.74713        0.00319
H        -3.70757       -1.52529       -0.90393
H        -3.69958       -1.54431        0.88321
O         1.69563        0.72752       -1.22008
S         2.02446       -0.08378        0.00092
C         3.80219       -0.30360       -0.00925
O         1.69893        0.65415        1.26734
O         1.41554       -1.44948       -0.03902
H         4.07053       -0.89137        0.87553
H         4.06668       -0.83293       -0.93147
H         4.25900        0.69197        0.02238
H        -1.54401        2.23179       -0.82839
H        -0.05922        1.62107        0.00038
H        -1.48497        2.15709        0.97183
24
TMEA-NO3-p1.log        Energy: -334947.1187297
C        -0.75685        1.08952       -0.60935
N        -1.25216       -0.05922        0.21405
C        -2.72123        0.06061        0.50076
C        -3.61095        0.18688       -0.72099
C        -0.93612       -1.33593       -0.49856
C        -0.50743       -0.05362        1.51589
H        -1.46176       -1.34368       -1.45948
H         0.15712       -1.36228       -0.62818
H        -1.14470        2.01677       -0.16684
H         0.34655        1.08372       -0.55503
H        -2.82646        0.94134        1.15024
H        -2.98622       -0.82764        1.09133
H        -1.12560        0.97400       -1.63433
H        -1.27242       -2.17042        0.12993
H        -4.65167        0.25501       -0.37645
H        -3.39313        1.09457       -1.29835
H        -3.54193       -0.68579       -1.38374
O         1.99422       -1.02069       -0.03549
N         2.69382        0.03169       -0.10059
O         3.86977       -0.02320       -0.40676
O         2.13968        1.12914        0.16660
H        -0.65308        0.92643        1.98553
H         0.55240       -0.22703        1.28378
H        -0.92466       -0.84910        2.14609
27
TMEA-TFA-p1.log        Energy: -489226.6053900
C        -2.22833       -1.72894       -0.34728
N        -2.57938       -0.33464        0.06587
C        -1.94486        0.61076       -0.94349
C        -1.87814        2.05459       -0.48957
C        -4.05401       -0.17417        0.07315
C        -2.03780       -0.09013        1.44105
H        -4.44646       -0.45171       -0.91268
H        -4.47523       -0.83193        0.84338
```

```
H        -1.13076      -1.80887      -0.30995
H        -2.72827      -2.42692       0.33575
H        -2.54584       0.48837      -1.85705
H        -0.93582       0.20682      -1.10565
H        -2.58860      -1.87868      -1.37302
H        -4.30230       0.86902       0.29838
H        -1.54227       2.65119      -1.34892
H        -2.84986       2.46109      -0.17019
H        -1.12284       2.16617       0.29860
F         3.20911       1.09290      -0.60398
F         3.34013      -1.05998      -0.69796
C         2.80882      -0.04554      -0.00892
C         1.26180      -0.10766       0.07087
O         0.75218       0.80029       0.75686
O         0.71406      -1.03481      -0.55918
F         3.36037      -0.06222       1.21567
H        -2.48649       0.83133       1.83018
H        -0.94397       0.03473       1.36164
H        -2.32682      -0.94176       2.06865
```

1.2 Solvent geometries

```
25
DMEA-CF3SO3-p1.log        Energy: -737684.9953746
C         2.39909      -0.44932       1.37306
N         2.44463       0.09738      -0.00829
C         3.55998       1.07245      -0.22471
C         4.94042       0.45778      -0.09710
C         2.41059      -0.99026      -1.02027
H         1.53381       0.60453      -0.12669
H         3.22641      -1.69541      -0.82775
H         1.44041      -1.49678      -0.94666
H         2.50005       0.38168       2.08028
H         1.42636      -0.93700       1.51294
H         3.41052       1.87158       0.51241
H         3.40407       1.49586      -1.22525
H         3.21390      -1.16847       1.50962
H         2.51681      -0.54508      -2.01596
H         5.68270       1.24981      -0.25988
H         5.11422       0.03933       0.90329
H         5.11540      -0.32212      -0.85001
O        -0.15711       0.80756      -0.17139
S        -1.04629      -0.38295       0.03875
C        -2.72942       0.37582      -0.05201
O        -1.02440      -1.35946      -1.07235
O        -0.98248      -0.95191       1.40370
F        -3.66102      -0.56158       0.11810
F        -2.87674       1.29716       0.89813
F        -2.92095       0.95322      -1.23641
18
```

```
DMEA-Cl-p1.log        Energy: -423264.2431468
C          0.56981        1.12017       -1.22919
N          0.31997        0.33738        0.00010
C          1.00111       -0.99138        0.00707
C          2.51634       -0.91898        0.00032
C          0.56783        1.13194        1.22226
H         -0.73578        0.09951        0.00034
H          1.61238        1.46401        1.24910
H         -0.09825        2.00203        1.20674
H          0.35155        0.48773       -2.09739
H         -0.10016        1.98739       -1.22546
H          0.62208       -1.52547       -0.87486
H          0.62907       -1.51264        0.89951
H          1.61300        1.45656       -1.25487
H          0.34290        0.50957        2.09599
H          2.90942       -1.94403        0.00972
H          2.90137       -0.42386       -0.90138
H          2.90870       -0.40474        0.88810
Cl        -2.53686       -0.38872        0.00006
26
DMEA-mOSO3-p1.log        Energy: -598092.5007628
C          1.63266        1.40357        0.57420
N          1.84079        0.01573        0.09396
C          2.92330       -0.10032       -0.93157
C          4.30079        0.28142       -0.42424
C          1.99963       -0.92777        1.22712
H          0.93850       -0.25628       -0.39876
H          2.84978       -0.62011        1.84682
H          1.06895       -0.90255        1.80419
H          1.52610        2.06244       -0.29561
H          0.71211        1.40750        1.16792
H          2.61716        0.54008       -1.76947
H          2.90308       -1.14275       -1.27585
H          2.48859        1.71477        1.18387
H          2.16809       -1.93314        0.82419
H          5.01784        0.14741       -1.24454
H          4.34721        1.33271       -0.11026
H          4.62458       -0.35756        0.40842
H         -3.83771        2.21963       -0.35739
O         -2.30876        0.91897       -0.64799
O         -0.46964       -0.59221       -1.09504
C         -3.47998        1.28372        0.08556
H         -4.24843        0.50356       -0.00870
S         -1.58440       -0.45432       -0.10047
O         -2.59821       -1.52122       -0.16604
H         -3.23496        1.44166        1.14630
O         -1.09018       -0.13583        1.26434
25
DMEA-mSO3-p1.log        Energy: -550905.6140174
C          1.49459        1.02173        1.13292
N          1.53560       -0.02041        0.08070
C          2.58062        0.22357       -0.95632
C          4.00398        0.15807       -0.43443
C          1.59510       -1.37807        0.67080
```

```
H          0.58385        0.03456       -0.42826
H          2.47111       -1.46321        1.32490
H          0.66943       -1.52170        1.23958
H          1.44887        2.00520        0.64978
H          0.58740        0.84803        1.72228
H          2.36087        1.21137       -1.38361
H          2.41275       -0.52796       -1.73930
H          2.38826        0.95234        1.76470
H          1.65719       -2.11117       -0.14225
H          4.68756        0.35678       -1.27023
H          4.19403        0.91487        0.33846
H          4.24842       -0.83458       -0.03239
O         -0.79297        0.08184       -1.10780
S         -1.91545       -0.13354       -0.09974
C         -2.78590        1.42500       -0.05414
O         -2.88535       -1.16410       -0.54410
O         -1.35716       -0.34842        1.27280
H         -3.59503        1.33454        0.67944
H         -2.07529        2.20491        0.24228
H         -3.18807        1.61918       -1.05482
21
DMEA-NO3-p1.log        Energy: -310325.1093906
C          0.97298        0.96646       -1.22317
N          0.92641        0.13430        0.00013
C          1.92581       -0.97534        0.00095
C          3.37135       -0.51536       -0.00018
C          0.97364        0.96896        1.22165
H         -0.04191       -0.34788        0.00073
H          1.87834        1.58827        1.21752
H          0.07647        1.59676        1.22180
H          0.96888        0.30615       -2.09820
H          0.07575        1.59418       -1.22413
H          1.70235       -1.58306       -0.88625
H          1.70312       -1.58113        0.88967
H          1.87764        1.58583       -1.22069
H          0.96988        0.31048        2.09806
H          4.01602       -1.40393        0.00067
H          3.61550        0.07209       -0.89550
H          3.61618        0.07429        0.89351
O         -1.40043       -1.08711        0.00099
N         -2.28923       -0.17761        0.00020
O         -3.47077       -0.49479       -0.00033
O         -1.92645        1.00976       -0.00005
24
DMEA-TFA-p1.log        Energy: -464601.6835228
C         -2.27344       -1.70795       -0.58337
N         -2.21803       -0.41068        0.12516
C         -2.41788        0.71748       -0.83046
C         -2.19130        2.06907       -0.17691
C         -3.15279       -0.37687        1.26871
H         -1.19168       -0.29580        0.50340
H         -4.18554       -0.42102        0.89711
H         -2.95037       -1.24124        1.91086
H         -1.48133       -1.70702       -1.34038
```

```
H         -2.10002       -2.51186        0.14113
H         -3.43040        0.62216       -1.25069
H         -1.67989        0.55025       -1.62465
H         -3.26167       -1.82808       -1.04649
H         -2.99782        0.54404        1.84011
H         -2.16177        2.83974       -0.95825
H         -2.99189        2.33535        0.52571
H         -1.22846        2.07847        0.35513
F          3.15361        0.00840       -1.04076
F          2.91513       -0.95332        0.87812
C          2.43838        0.02415        0.08800
C          0.92307       -0.16367       -0.16299
O          0.55573       -0.37428       -1.31898
O          0.24125       -0.08168        0.89850
F          2.69292        1.18761        0.70895
22
EtMeNH2-CF3SO3-p1.log         Energy: -713031.9891680
C         -2.56864       -1.64654        0.16825
N         -1.96172       -0.32360       -0.11206
C         -2.88320        0.73158       -0.61826
C         -3.96455        1.08481        0.38362
H         -1.47385       -0.00798        0.73865
H         -1.17339       -0.48008       -0.78372
H         -3.06637       -2.00145       -0.74111
H         -1.75368       -2.31923        0.45389
H         -3.28962       -1.55610        0.98648
H         -4.53891        1.93666       -0.00239
H         -4.66431        0.25466        0.54669
H         -3.52730        1.37933        1.34897
H         -3.30364        0.35546       -1.55992
H         -2.25120        1.59906       -0.84503
O          0.44545       -0.99786       -1.27374
S          1.24174       -0.86275       -0.01138
C          1.53048        0.96288        0.04731
O          0.42028       -1.08179        1.20985
O          2.57952       -1.47304       -0.03423
F          2.18265        1.31284        1.15187
F          2.21865        1.38367       -1.00872
F          0.33868        1.58504        0.04836
15
EtMeNH2-Cl-p1.log         Energy: -398609.5691512
C          0.56954        1.62614       -0.20280
N          0.39978        0.34272        0.51101
C          1.12284       -0.81368       -0.08567
C          2.62783       -0.63277       -0.10395
H          0.66057        0.44906        1.49724
H         -0.65195        0.09883        0.46864
H          0.28947        1.46806       -1.25109
H         -0.10164        2.36322        0.25018
H          1.60708        1.96812       -0.13214
H          3.09027       -1.55477       -0.47958
H          2.93649        0.18970       -0.76270
H          3.01704       -0.44511        0.90739
H          0.70920       -0.93749       -1.09577
```

```
H          0.83204       -1.69049        0.50640
Cl        -2.41813       -0.31684       -0.06654
23
EtMeNH2-mOSO3-p1.log        Energy: -573439.1456032
C         -3.34455        0.99807        0.14199
N         -1.98312        0.48512        0.39981
C         -1.87143       -1.00128        0.52373
C         -1.96714       -1.67742       -0.82995
H         -1.30289        0.77818       -0.34907
H         -1.58047        0.90657        1.24628
H         -4.00018        0.69810        0.96657
H         -3.30241        2.08934        0.07035
H         -3.71144        0.58049       -0.80098
H         -2.94672       -1.52818       -1.30451
H         -1.17541       -1.29924       -1.49177
H         -1.82145       -2.75734       -0.69634
H         -2.65636       -1.33027        1.21761
H         -0.89057       -1.18304        0.97935
O          0.18998        0.88543       -1.18277
O          1.42473       -1.02084       -0.33599
S          1.21916        0.60679       -0.13177
O          2.53271        1.22950       -0.36603
H          2.42911       -2.66303        0.30508
O          0.68034        0.76239        1.24719
C          2.39148       -1.59513        0.54625
H          3.37723       -1.13674        0.38214
H          2.08458       -1.45653        1.59372
22
EtMeNH2-mSO3-p1.log        Energy: -526250.6613066
C         -1.56249        1.46540        0.06880
N         -1.52882       -0.00835        0.21252
C         -2.82245       -0.66258        0.53556
C         -3.86539       -0.45102       -0.54569
H         -1.08752       -0.40129       -0.63992
H         -0.78107       -0.22824        0.91698
H         -1.99854        1.89691        0.97737
H         -0.53012        1.81313       -0.05825
H         -2.16120        1.73924       -0.80554
H         -4.75838       -1.03838       -0.29663
H         -4.16742        0.60168       -0.62506
H         -3.49531       -0.79119       -1.52381
H         -3.15344       -0.25593        1.50047
H         -2.60330       -1.72887        0.67026
O          0.93357       -0.24787        1.27428
S          1.62539        0.01494       -0.04581
C          3.08699       -1.00634       -0.03558
H          3.61323       -0.84581       -0.98312
H          3.70736       -0.69930        0.81360
H          2.76958       -2.04994        0.06532
O          0.78235       -0.46220       -1.19779
O          2.07429        1.42691       -0.18960
18
EtMeNH2-NO3-p1.log        Energy: -285670.0049643
C         -1.05920        1.34030        0.70058
```

```
N         -0.97399        0.08005       -0.06856
C         -2.12902       -0.84326        0.07337
C         -3.43099       -0.24391       -0.42339
H         -0.80953        0.30628       -1.05602
H         -0.05125       -0.39744        0.23407
H         -1.23778        1.09197        1.75338
H         -0.09747        1.85144        0.58637
H         -1.87069        1.96883        0.31927
H         -4.21923       -1.00560       -0.36519
H         -3.74681        0.61361        0.18546
H         -3.34354        0.07718       -1.47167
H         -2.18759       -1.11015        1.13722
H         -1.87033       -1.74830       -0.49029
O          1.38483       -0.82206        0.64410
N          2.14444       -0.11200       -0.08813
O          3.35538       -0.27538       -0.03103
O          1.62934        0.72958       -0.84296
21
EtMeNH2-TFA-p1.log         Energy: -439945.9035235
C         -3.30220       -1.35571        0.51182
N         -2.23098       -0.65290       -0.21852
C         -2.59339        0.68926       -0.75202
C         -2.79806        1.69238        0.36829
H         -1.32678       -0.51103        0.38381
H         -1.88888       -1.22132       -1.00101
H         -4.18078       -1.46233       -0.13583
H         -2.93572       -2.34314        0.81133
H         -3.56634       -0.78006        1.40490
H         -3.67775        1.45566        0.98172
H         -1.90765        1.72564        1.01240
H         -2.95025        2.68910       -0.06552
H         -3.49008        0.57712       -1.37738
H         -1.75107        0.98037       -1.39213
O          0.37915       -0.42707       -1.32904
F          2.46456        1.20496        0.70248
F          2.95944        0.07104       -1.06797
C          2.25573        0.03994        0.06752
C          0.74794       -0.21171       -0.17226
O          0.06758       -0.17474        0.89146
F          2.78247       -0.92602        0.83970
19
EtNH3-CF3SO3-p1.log         Energy: -688379.2393931
C          3.24880       -0.98141       -0.02331
C          3.61535        0.48969        0.02522
N          2.38070        1.32550       -0.00930
H          1.73193        1.06524        0.76318
H          2.58131        2.32850        0.02520
H          1.80960        1.11260       -0.84718
H          4.22973        0.78726       -0.83247
H          4.15148        0.74812        0.94585
H          4.16580       -1.58338       -0.00254
H          2.62504       -1.25612        0.83857
H          2.69178       -1.21516       -0.94112
O          0.31426       -0.01133        1.23968
```

```
S         -0.33894       -0.54654        0.00418
C         -1.96661        0.32982       -0.00436
O          0.30890       -0.06571       -1.25208
O         -0.68854       -1.97627        0.04106
F         -2.68934       -0.03971       -1.06031
F         -2.65319        0.04487        1.10093
F         -1.77866        1.64783       -0.05905
12
EtNH3-Cl-p1.log        Energy: -373955.9924557
C          1.62988       -1.11569       -0.21427
C          1.55791        0.22769        0.48452
N          0.66068        1.14585       -0.26953
H         -0.34610        0.74647       -0.24078
H          0.64442        2.08641        0.13592
H          0.95210        1.23822       -1.24815
H          2.54208        0.70397        0.56736
H          1.12443        0.13529        1.48812
H          2.26008       -1.79740        0.37078
H          0.62476       -1.54963       -0.30572
H          2.07117       -1.01550       -1.21654
Cl        -1.97791       -0.19063        0.04201
20
EtNH3-mOSO3-p1.log        Energy: -548785.4817981
C          2.47088        1.32495       -0.24362
C          2.38942        0.06110        0.58829
N          2.11912       -1.11480       -0.29054
H          1.25304       -0.90987       -0.86886
H          2.90986       -1.33208       -0.90412
H          1.89003       -1.94411        0.26727
H          1.54958        0.10111        1.29175
H          3.31519       -0.14020        1.14020
H          2.64364        2.18412        0.41721
H          3.29626        1.27698       -0.96851
H          1.52538        1.47965       -0.78221
O         -0.39385       -1.41376        0.95563
O         -0.86123        0.95843        0.71513
S         -1.05037       -0.51160       -0.01664
O         -0.26537       -0.38914       -1.29596
H         -1.21447        2.95050        0.57137
O         -2.49549       -0.69397       -0.22908
C         -1.44702        2.04653       -0.00215
H         -1.01280        2.12131       -1.01058
H         -2.53575        1.91377       -0.07441
19
EtNH3-mSO3-p1.log        Energy: -501598.1001146
C         -2.22191        1.28668       -0.00000
C         -2.90066       -0.07031       -0.00001
N         -1.86935       -1.14174        0.00000
H         -1.20551       -1.02400        0.79917
H         -2.26898       -2.08298        0.00004
H         -1.20552       -1.02405       -0.79921
H         -3.52512       -0.21525       -0.88988
H         -3.52512       -0.21523        0.88988
H         -2.98137        2.07868        0.00005
```

```
H         -1.58652        1.39648        0.89014
H         -1.58656        1.39653       -0.89017
O          0.39700       -0.32216        1.24488
S          1.23369       -0.13583       -0.00000
C          1.71874        1.58193        0.00003
O          2.48238       -0.93796       -0.00004
O          0.39696       -0.32208       -1.24487
H          2.31490        1.76178       -0.90198
H          0.81349        2.19957        0.00005
H          2.31491        1.76176        0.90204
15
EtNH3-NO3-p1.log        Energy: -261016.9696111
C         -1.72868       -1.31808       -0.16343
C         -2.35735        0.06241       -0.17477
N         -1.37273        1.07355        0.29293
H         -0.44771        0.98236       -0.26903
H         -1.73996        2.02706        0.24175
H         -1.08154        0.88736        1.25907
H         -3.23874        0.11731        0.47561
H         -2.65666        0.35657       -1.18830
H         -2.46042       -2.05353       -0.52124
H         -0.84981       -1.34220       -0.82225
H         -1.41233       -1.60016        0.85008
O          0.90774        0.59592       -0.91247
N          1.56347        0.01090        0.01065
O          2.70762       -0.35835       -0.21357
O          1.01816       -0.16657        1.11085
18
EtNH3-TFA-p1.log        Energy: -415291.9579503
C          4.26821        0.60729        0.22508
C          2.89507        0.05623        0.55336
N          2.34312       -0.76964       -0.54870
H          2.22974       -0.20032       -1.39420
H          2.94622       -1.56554       -0.77307
H          1.30672       -1.08920       -0.27429
H          2.16286        0.85734        0.71489
H          2.91777       -0.57950        1.44726
H          4.63213        1.21327        1.06515
H          4.99045       -0.20297        0.04938
H          4.23383        1.24677       -0.66859
O          0.03209        0.90610       -0.73604
F         -2.54595        1.28380       -0.30228
F         -2.30385       -0.03217        1.39322
C         -2.06113        0.09846        0.07803
C         -0.55154       -0.04906       -0.22443
O         -0.09235       -1.17996        0.11081
F         -2.77721       -0.84929       -0.54829
21
mim-CF3SO3-p1.log        Energy: -770161.8274858
C          3.63623        0.96078        0.41679
N          2.41072        0.73481       -0.17540
C          2.32091       -0.54655       -0.51818
N          3.45927       -1.14308       -0.16604
C          4.30186       -0.22778        0.42266
```

```
C          1.34743        1.72054       -0.35532
H          3.66042       -2.12675       -0.31621
H          1.44831       -0.99953       -0.98745
H          5.28655       -0.49624        0.78482
H          3.92612        1.94148        0.77613
H          1.77780        2.62454       -0.79949
H          0.59305        1.28273       -1.01558
H          0.89331        1.93733        0.61721
O         -0.61227       -0.81393       -1.32624
S         -1.17051       -0.85837        0.05768
O         -2.00777       -2.03695        0.36191
O         -0.20093       -0.45897        1.10867
C         -2.38461        0.53892        0.04614
F         -2.96988        0.66221        1.23829
F         -1.77549        1.69562       -0.23878
F         -3.33359        0.33457       -0.86834
21
mim-CF3SO3-p2.log        Energy: -770165.3626859
C          3.94985        0.87606        0.40032
N          3.56739       -0.41253        0.09065
C          2.25964       -0.41827       -0.17442
N          1.79701        0.82082       -0.04526
C          2.83052        1.65071        0.31305
C          4.44853       -1.57546        0.05302
H          0.78891        1.08758       -0.20781
H          1.65314       -1.27539       -0.45092
H          2.69020        2.71252        0.47576
H          4.97516        1.12269        0.65162
H          4.90339       -1.71743        1.03917
H          3.85192       -2.45350       -0.21054
H          5.22699       -1.41590       -0.70083
O         -0.78534        1.35787       -0.53361
S         -1.47301        0.05107       -0.80880
O         -2.46715        0.09218       -1.89425
O         -0.54122       -1.10349       -0.79533
C         -2.47997       -0.18467        0.72213
F         -3.15510       -1.33250        0.66451
F         -1.68960       -0.21273        1.79534
F         -3.35243        0.81206        0.86545
14
mim-Cl-p1.log        Energy: -455741.4270486
C          1.65454        1.17277        0.00003
N          1.79532       -0.19780       -0.00002
C          0.57660       -0.74706        0.00019
N         -0.32948        0.22121        0.00002
C          0.31372        1.43212        0.00001
C          3.05722       -0.93620       -0.00010
H         -1.39649        0.05696       -0.00000
H          0.36927       -1.81232        0.00028
H         -0.22734        2.37078       -0.00004
H          2.51286        1.83469       -0.00000
H          3.87644       -0.21152       -0.00040
H          3.11688       -1.55984        0.89826
H          3.11655       -1.56022       -0.89822
```

```
Cl        -3.24950       -0.28307       -0.00004
14
mim-Cl-p2.log        Energy: -455736.9201602
C          2.16575        0.62384        0.00003
N          0.78629        0.66174        0.00003
C          0.31159       -0.58202        0.00003
N          1.35602       -1.41149       -0.00002
C          2.52721       -0.68926       -0.00003
C         -0.04387        1.86616       -0.00002
H          1.28246       -2.42407       -0.00009
H         -0.75832       -0.83017        0.00001
H          3.50097       -1.16360        0.00007
H          2.76439        1.52727       -0.00004
H          0.18045        2.45310       -0.89760
H         -1.09305        1.54506        0.00004
H          0.18051        2.45327        0.89743
Cl        -2.98928       -0.33088        0.00000
22
mim-mOSO3-p1.log        Energy: -630566.4154394
C          3.17812        0.71519       -0.32309
N          1.92065        0.63997        0.24138
C          1.65671       -0.62612        0.55372
N          2.71576       -1.35831        0.20876
C          3.68315       -0.54940       -0.34338
C          0.99483        1.75603        0.42667
H          2.78166       -2.36299        0.33887
H          0.71841       -0.96896        0.99272
H          4.63165       -0.93608       -0.69506
H          3.60108        1.65629       -0.65589
H          1.55332        2.60865        0.82766
H          0.53793        2.01192       -0.53613
H          0.21281        1.44244        1.12471
H         -1.61446        1.05369       -2.19392
O         -1.17740       -0.54035        1.53815
H         -2.20729       -0.62932       -2.41630
C         -1.33658       -0.01036       -2.15379
S         -1.98091       -0.11611        0.35842
O         -0.85872       -0.35109       -0.85544
H         -0.51466       -0.20855       -2.85159
O         -2.28796        1.33243        0.31998
O         -3.11571       -1.00261        0.02463
21
mim-mSO3-p1.log        Energy: -583384.0707582
C         -3.26832        0.95232       -0.04489
N         -2.89724       -0.37552       -0.02067
C         -1.56284       -0.44273        0.01838
N         -1.07049        0.78981        0.01969
C         -2.11407        1.68084       -0.01914
C         -3.80762       -1.51481       -0.03303
H         -0.00247        1.00694        0.05040
H         -0.95336       -1.34121        0.04326
H         -1.95915        2.75319       -0.02509
H         -4.30898        1.25483       -0.07699
H         -4.45258       -1.47643        0.85172
```

```
H         -4.41782       -1.48445       -0.94228
H         -3.21280       -2.43280       -0.01741
O          1.24021       -1.25367        0.02861
S          2.20950       -0.12118        0.14133
O          1.48005        1.21661        0.09960
O          3.14902       -0.21508        1.28528
C          3.19215       -0.14030       -1.34755
H          3.87682        0.71452       -1.31438
H          3.74947       -1.08381       -1.37009
H          2.51297       -0.06676       -2.20413
21
mim-mSO3-p2.log        Energy: -583376.8396183
C         -2.89943        0.44495        0.62007
N         -1.78563        0.70087       -0.15518
C         -1.39723       -0.42657       -0.74450
N         -2.23607       -1.39180       -0.37111
C         -3.18499       -0.87935        0.48419
C         -1.12913        1.99986       -0.30452
H         -2.15962       -2.36042       -0.66469
H         -0.51385       -0.52140       -1.37293
H         -3.96874       -1.49124        0.91335
H         -3.38829        1.22549        1.19155
H         -1.84446        2.71156       -0.73138
H         -0.77797        2.32887        0.67852
H         -0.25986        1.85767       -0.95442
O          1.41890        0.95463        1.09806
S          1.74538       -0.09935        0.09017
O          1.14773       -1.43402        0.40724
O          1.44457        0.33351       -1.32279
C          3.52236       -0.31841        0.15040
H          3.79556       -1.08567       -0.58295
H          3.99270        0.64080       -0.09440
H          3.79124       -0.63539        1.16460
17
mim-NO3-p1.log        Energy: -342803.3893619
C         -2.55746        1.01917       -0.00210
N         -2.29026       -0.33202        0.00042
C         -0.96278       -0.49994        0.00206
N         -0.37781        0.69033        0.00059
C         -1.35025        1.65838       -0.00183
C         -3.28121       -1.40536        0.00124
H          0.70973        0.82897        0.00161
H         -0.43525       -1.44786        0.00409
H         -1.11397        2.71585       -0.00324
H         -3.57236        1.39983       -0.00382
H         -4.27609       -0.95027       -0.00098
H         -3.15732       -2.02251       -0.89535
H         -3.15968       -2.01891        0.90062
O          2.19162        0.97694        0.00385
N          2.67649       -0.20032       -0.00032
O          3.89163       -0.34343        0.00000
O          1.89878       -1.16408       -0.00435
17
mim-NO3-p2.log        Energy: -342795.4870053
```

```
C         -1.34221        0.31971       -1.08028
N         -0.83121        0.90758        0.05737
C         -1.07209        0.11435        1.09615
N         -1.74867       -0.94478        0.66038
C         -1.92417       -0.85119       -0.70007
C         -0.06962        2.15596        0.09588
H         -2.04238       -1.71842        1.24793
H         -0.75420        0.29532        2.11665
H         -2.44115       -1.61256       -1.27027
H         -1.24017        0.78275       -2.05446
H         -0.61770        2.91309       -0.47505
H          0.02312        2.47117        1.13923
H          0.91945        1.96084       -0.33313
O          1.91397        0.12688        0.98487
N          1.63992       -0.49725       -0.06054
O          1.83955        0.03522       -1.17057
O          1.14415       -1.63510        0.00551
20
mim-TFA-p1.log        Energy: -497071.0980654
C          4.09599       -0.41337        0.01822
N          2.73796       -0.65530       -0.01788
C          2.08168        0.50393       -0.01752
N          2.99471        1.47741        0.01795
C          4.25985        0.93873        0.04131
C          2.10645       -1.97427       -0.05685
H          2.76934        2.46729        0.02634
H          0.98746        0.64788       -0.04358
H          5.15193        1.55195        0.07051
H          4.81986       -1.22028        0.02448
H          2.43364       -2.54850        0.81737
H          1.01970       -1.82316       -0.03662
H          2.41375       -2.48809       -0.97489
F         -3.49022       -0.51822        1.09324
O         -0.84848        1.13891       -0.08791
C         -1.42525        0.03905        0.00551
F         -3.48290       -0.55293       -1.06552
O         -0.96807       -1.11314        0.09160
C         -2.97795        0.08884        0.00623
F         -3.47538        1.33275       -0.01606
20
mim-TFA-p2.log        Energy: -497062.7374311
C          3.53966        0.87282        0.69326
N          2.54847        0.25325       -0.03821
C          3.07836       -0.77071       -0.69815
N          4.37863       -0.82313       -0.40949
C          4.69760        0.19254        0.45955
C          1.13985        0.65760       -0.08344
H          5.02307       -1.51345       -0.78337
H          2.54424       -1.44633       -1.35791
H          5.70363        0.34105        0.83210
H          3.33264        1.73803        1.31270
H          0.58926       -0.01948       -0.73852
H          0.71294        0.60400        0.92222
H          1.06546        1.67958       -0.46499
```

```
F         -3.41061       -1.32199       -0.55838
O         -3.73775        1.38235       -0.44200
C         -2.59099        0.93643       -0.28866
F         -2.95828       -0.67837        1.45068
O         -1.47465        1.47216       -0.40702
C         -2.57005       -0.55593        0.16233
F         -1.35648       -1.13594        0.07819
20
mim-TFA-p3.log        Energy: -497067.1959731
C          1.68973       -0.42732       -0.14924
N          3.06139       -0.31651       -0.03374
C          3.37670        0.96519        0.12922
N          2.24584        1.66924        0.11960
C          1.16909        0.82998       -0.05390
C          4.01016       -1.42584       -0.08912
H          2.20131        2.67840        0.22437
H          4.37812        1.36448        0.24837
H          0.13535        1.18950       -0.08979
H          1.20139       -1.38465       -0.28840
H          5.02166       -1.02713        0.03052
H          3.78759       -2.12906        0.72029
H          3.91845       -1.92622       -1.05904
F         -1.19573       -0.69919        1.16640
O         -3.85737        0.80900       -0.10303
C         -2.62312        0.78578       -0.08307
F         -2.84435       -1.63113        0.12610
O         -1.77754        1.70286       -0.15835
C         -1.96889       -0.62207        0.06110
F         -1.14184       -0.88787       -0.97986
26
mpyr-CF3SO3-p1.log        Energy: -761597.2903892
C          3.05199       -0.06895       -1.12003
N          2.44523        0.79426       -0.06358
C          2.49958       -0.03022        1.18692
C          2.31094       -1.48004        0.71191
C          2.44461       -1.43521       -0.82847
C          3.03731        2.14042        0.06348
H          1.42725        0.87996       -0.31700
H          2.52234        2.67604        0.86809
H          4.10341        2.03818        0.29948
H          2.90893        2.67330       -0.88485
H          2.81057        0.34635       -2.10557
H          4.14091       -0.05162       -0.96874
H          1.45152       -1.50758       -1.29146
H          3.07370       -2.24009       -1.22506
H          1.32273       -1.85660        1.00397
H          3.07293       -2.12342        1.16762
H          1.70988        0.32023        1.86103
H          3.48596        0.13888        1.63795
O         -0.15706        0.58390       -0.78195
S         -0.94027       -0.33405        0.11390
C         -2.66407        0.29913       -0.10171
O         -1.00619       -1.73159       -0.36269
O         -0.65823       -0.14588        1.55534
```

F	-3.51151	-0.41919	0.63366
F	-2.74644	1.57184	0.28008
F	-3.03236	0.21282	-1.37829

19

mpyr-Cl-p1.log Energy: -447174.7923491

C	0.96760	0.19424	-1.18270
N	0.25114	0.71712	0.03144
C	0.87863	0.07858	1.22333
C	1.20724	-1.31257	0.70776
C	1.76410	-1.02597	-0.69306
C	0.12925	2.18438	0.09836
H	-0.74502	0.31098	-0.01558
H	-0.45713	2.44649	0.98646
H	1.12932	2.63127	0.15919
H	-0.38839	2.53451	-0.80206
H	0.19652	-0.05406	-1.92285
H	1.60699	0.99062	-1.58178
H	1.65845	-1.87841	-1.37370
H	2.83064	-0.77166	-0.62859
H	0.27962	-1.90004	0.64519
H	1.91795	-1.83825	1.35575
H	0.16259	0.11085	2.05355
H	1.78161	0.64902	1.48476
Cl	-2.43600	-0.52724	-0.08839

27

mpyr-mOSO3-p1.log Energy: -622002.5500045

C	1.83007	0.11574	-1.25701
N	1.78864	0.85756	0.06025
C	2.15289	-0.13109	1.14521
C	2.77645	-1.30527	0.40385
C	1.94154	-1.35586	-0.87588
C	2.61402	2.08518	0.06263
H	0.77836	1.11430	0.22155
H	2.44516	2.62413	1.00127
H	3.67069	1.80492	-0.02946
H	2.31556	2.70973	-0.78672
H	0.92117	0.36345	-1.81773
H	2.71804	0.46157	-1.80178
H	0.94252	-1.76460	-0.66690
H	2.40495	-1.94133	-1.67905
H	2.72569	-2.22614	0.99692
H	3.83208	-1.09998	0.17015
H	1.20831	-0.42649	1.62070
H	2.80501	0.36081	1.87486
O	-0.99274	-1.34870	1.03427
O	-3.03160	-0.12640	0.49274
O	-1.37437	-0.61241	-1.32791
S	-1.44216	-0.28443	0.11442
H	-4.76358	0.83677	0.06616
H	-3.69778	0.56532	-1.35719
C	-3.72925	0.84281	-0.29427
O	-0.84883	1.06470	0.42339
H	-3.28625	1.83893	-0.15245

26

```
mpyr-mSO3-p1.log         Energy: -574816.7741667
C          2.26375       -0.25365       -1.07384
N          1.70768        0.68535       -0.06000
C          1.71763       -0.08601        1.21952
C          1.39091       -1.52811        0.80344
C          1.56431       -1.56492       -0.73448
C          2.37453        1.99676        0.00945
H          0.67674        0.82190       -0.34138
H          1.89283        2.59659        0.78968
H          3.43542        1.85014        0.24915
H          2.27385        2.49709       -0.96028
H          2.05276        0.13604       -2.07709
H          3.35166       -0.30396       -0.91897
H          0.57513       -1.59046       -1.21079
H          2.14685       -2.42669       -1.07994
H          0.35620       -1.77434        1.07157
H          2.06426       -2.22889        1.31165
H          0.97122        0.35360        1.88967
H          2.72513        0.01145        1.64676
O         -0.77890        0.79281       -0.89413
S         -1.72315        0.00437        0.00548
C         -3.31718        0.76561       -0.25191
O         -1.84560       -1.41671       -0.42350
O         -1.37711        0.18536        1.44591
H         -4.04414        0.22658        0.36595
H         -3.24556        1.81563        0.05202
H         -3.56650        0.67819       -1.31508
22
mpyr-NO3-p1.log        Energy: -334235.4175201
C          1.34893       -0.27754       -1.17393
N          1.06148        0.70188       -0.07938
C          1.60184        0.08752        1.16438
C          1.17839       -1.36521        1.01215
C          1.39211       -1.65332       -0.48618
C          1.53095        2.07279       -0.34798
H         -0.00485        0.75432        0.07189
H          1.22847        2.71811        0.48484
H          2.62387        2.07032       -0.44560
H          1.07141        2.42799       -1.27750
H          0.55733       -0.18434       -1.92409
H          2.31541       -0.00026       -1.61569
H          0.60786       -2.31125       -0.87576
H          2.36725       -2.12616       -0.65944
H          0.11220       -1.45682        1.26032
H          1.75482       -2.02894        1.66665
H          1.17681        0.60677        2.03197
H          2.69522        0.20937        1.15667
O         -1.49166        0.98283        0.49840
N         -2.15257        0.02671       -0.01719
O         -3.37051        0.00836        0.10073
O         -1.53553       -0.86177       -0.62523
25
mpyr-TFA-p1.log        Energy: -488510.1252433
C          1.65505        0.63891       -1.07404
```

N	1.60527	0.58784	0.41297
C	2.64608	-0.41514	0.77488
C	2.46460	-1.52064	-0.27172
C	1.74943	-0.83527	-1.46497
C	1.73284	1.88999	1.08670
H	0.67200	0.10200	0.67624
H	1.69280	1.73292	2.17043
H	2.69057	2.34911	0.80898
H	0.90155	2.53018	0.77311
H	0.76507	1.15686	-1.44887
H	2.55554	1.20545	-1.35228
H	0.73924	-1.24460	-1.58705
H	2.28835	-0.95508	-2.41154
H	1.83667	-2.32181	0.13477
H	3.43525	-1.94596	-0.55175
H	2.48203	-0.73642	1.80996
H	3.62396	0.08051	0.69558
F	-3.15587	0.65726	-0.34538
O	-2.55714	-1.53338	1.05058
C	-1.59536	-0.85632	0.71365
F	-1.17604	1.42458	0.07246
O	-0.37104	-0.97079	1.01280
C	-1.87260	0.31432	-0.26788
F	-1.46528	-0.00736	-1.51543

28

TMEA-CF3SO3-p1.log Energy: -762334.2844849

C	2.26099	-0.45207	1.22751
N	2.73600	0.25639	0.00020
C	4.24228	0.37901	-0.00231
C	4.99218	-0.93788	-0.00003
C	2.25932	-0.47290	-1.21423
C	2.15923	1.63599	-0.01125
H	2.59259	-1.51368	-1.15785
H	1.16507	-0.42843	-1.23529
H	2.67510	0.06162	2.10289
H	1.16622	-0.41873	1.24235
H	4.49177	0.97613	0.88461
H	4.48877	0.97020	-0.89406
H	2.60501	-1.49030	1.19450
H	2.68304	0.01812	-2.09798
H	6.06444	-0.70194	0.00038
H	4.78564	-1.53586	0.89677
H	4.78650	-1.53767	-0.89580
O	-0.98170	0.78324	1.24397
S	-1.27558	0.02554	0.00026
C	-3.12171	-0.11249	-0.00044
O	-0.97862	0.76106	-1.25603
O	-0.81411	-1.38531	0.01350
F	-3.54109	-0.77812	-1.07707
F	-3.54255	-0.76167	1.08569
F	-3.68190	1.09749	-0.00988
H	2.51265	2.16473	0.88106
H	1.06662	1.55684	-0.00604
H	2.50545	2.14644	-0.91693

```
21
TMEA-Cl-p1.log        Energy: -447911.0777050
C          0.24562      -0.52176       1.20564
N          0.66840       0.25266      -0.00023
C          2.15713       0.49785      -0.00363
C          3.01374      -0.75289      -0.00034
C          0.23449      -0.48328      -1.22622
C         -0.02890       1.57534       0.02411
H          0.68861      -1.47924      -1.22291
H         -0.86071      -0.55829      -1.18573
H          0.59039       0.01382       2.09820
H         -0.85049      -0.58781       1.17741
H          2.35768       1.11289       0.88374
H          2.35424       1.10774      -0.89540
H          0.69219      -1.52020       1.16157
H          0.56266       0.08530      -2.10433
H          4.06329      -0.43049      -0.01376
H          2.86565      -1.35746       0.90354
H          2.84836      -1.37593      -0.88862
Cl        -3.12709      -0.25278       0.00006
H          0.27392       2.10813       0.93259
H         -1.10896       1.37747       0.02164
H          0.27232       2.14114      -0.86464
29
TMEA-mOSO3-p1.log        Energy: -622739.7978588
C          1.73689      -0.42503       1.23464
N          2.15635       0.30574       0.00022
C          3.64728       0.55090       0.00182
C          4.50264      -0.70047       0.00113
C          1.74690      -0.47289      -1.20801
C          1.46772       1.63297      -0.02883
H          2.17042      -1.47975      -1.14066
H          0.65294      -0.52367      -1.23125
H          2.09359       0.13938       2.10430
H          0.64371      -0.49331       1.24084
H          3.84496       1.16311       0.89167
H          3.84658       1.16326      -0.88747
H          2.17779      -1.42658       1.22079
H          2.12997       0.04530      -2.09520
H          5.55215      -0.37790       0.01073
H          4.33958      -1.31972       0.89242
H          4.35229      -1.30828      -0.90017
H         -4.05578       1.18942       0.89769
O         -3.42238      -0.58278       0.00379
O         -1.54481       0.51824      -1.24648
C         -4.24693       0.58195       0.00076
H         -4.05846       1.18292      -0.90115
S         -1.80151      -0.25311      -0.00044
O         -1.54277       0.53624       1.23381
H         -5.28329       0.22595       0.00353
O         -1.20731      -1.60899       0.00859
H          1.77801       2.20113       0.85513
H          0.38522       1.46439      -0.02074
H          1.77115       2.15761      -0.94178
```

```
28
TMEA-mSO3-p1.log        Energy: -575551.7724881
C          1.38705        0.45914       -1.22512
N          1.84231       -0.26661       -0.00024
C          3.34226       -0.43832        0.00069
C          4.13629        0.85285       -0.00018
C          1.38765        0.47463        1.21558
C          1.21848       -1.62585        0.00866
H          1.76274        1.50157        1.16429
H          0.29173        0.47045        1.22805
H          1.78107       -0.06930       -2.10144
H          0.29110        0.46388       -1.23144
H          3.57196       -1.04157       -0.88763
H          3.57084       -1.03891        0.89111
H          1.77090        1.48357       -1.19219
H          1.79129       -0.03648        2.09779
H          5.20040        0.58225       -0.00063
H          3.94880        1.45799       -0.89638
H          3.94950        1.45822        0.89601
O         -1.81294       -0.69529       -1.24191
S         -2.15982        0.07077        0.00029
C         -3.94362        0.24444       -0.00197
O         -1.81444       -0.67983        1.25218
O         -1.60156        1.46092       -0.00796
H         -4.23039        0.80331        0.89592
H         -4.22947        0.78961       -0.90855
H         -4.38203       -0.75984        0.00550
H          1.55591       -2.16364       -0.88470
H          0.12928       -1.50602        0.00398
H          1.55019       -2.14901        0.91279
24
TMEA-NO3-p1.log        Energy: -334970.3864420
C         -0.91514        1.22053       -0.54303
N         -1.33087        0.00389        0.21783
C         -2.81571        0.00264        0.48018
C         -3.68194       -0.01241       -0.76378
C         -0.91242       -1.21281       -0.54154
C         -0.61188        0.00550        1.52970
H         -1.33722       -1.16783       -1.54912
H          0.18483       -1.21731       -0.57080
H         -1.28093        2.10365       -0.00519
H          0.18179        1.22139       -0.58142
H         -3.01086        0.89778        1.08564
H         -3.00628       -0.88143        1.10313
H         -1.34859        1.17877       -1.54710
H         -1.28581       -2.09580       -0.00861
H         -4.72956       -0.00883       -0.43508
H         -3.52864        0.87611       -1.38972
H         -3.52763       -0.91608       -1.36732
O          2.21469       -1.08174        0.11454
N          2.81102       -0.00187       -0.09124
O          3.98582       -0.00326       -0.47112
O          2.21200        1.07970        0.09776
H         -0.90995        0.90250        2.08423
```

```
H          0.46314        0.00960        1.31434
H         -0.90282       -0.89504        2.08228
27
TMEA-TFA-p1.log        Energy: -489245.2460263
C          1.38340       -0.87231        1.40971
N          2.14764       -0.44389        0.19815
C          2.42263        1.03886        0.34429
C          3.13771        1.67146       -0.83290
C          3.41201       -1.22408        0.11153
C          1.30530       -0.69788       -1.00988
H          4.01810       -1.00849        0.99891
H          3.15628       -2.28888        0.07670
H          0.44549       -0.30652        1.41475
H          1.19148       -1.94830        1.33416
H          3.01688        1.13307        1.26317
H          1.43422        1.48981        0.50225
H          1.99320       -0.65441        2.29368
H          3.95173       -0.94278       -0.79777
H          3.30496        2.72746       -0.58166
H          4.12035        1.22191       -1.02702
H          2.53521        1.64439       -1.74981
F         -1.89703       -0.52674       -1.48828
F         -3.53994       -0.74938       -0.10222
C         -2.25388       -0.39363       -0.19058
C         -1.96042        1.04758        0.32374
O         -0.73252        1.29319        0.39032
O         -2.94399        1.74421        0.57848
F         -1.54096       -1.32347        0.48414
H          1.89948       -0.49243       -1.90618
H          0.43178       -0.03985       -0.93942
H          1.00029       -1.75065       -0.99747
```

2 INTERACTION ENERGY DATA

System	HF Energy	Correlation Energy	Total Interaction Energy	Suite
c1mim-bf4-p1-mp2	-345.0188653	-34.59261546	-379.6114808	IL174
c1mim-br-p1-mp2	-357.1522196	-45.4351599	-402.5873795	IL174
c1mim-br-p2-mp2	-353.9538208	-30.18904041	-384.1428612	IL174
c1mim-cl-p1-mp2	-378.4981773	-38.54406944	-417.0422467	IL174
c1mim-cl-p2-mp2	-377.970767	-27.53587869	-405.5066457	IL174
c1mim-dca-p1-mp2	-311.5039837	-55.62964364	-367.1336274	IL174
c1mim-dca-p2-mp2	-311.8887748	-42.80619942	-354.6949742	IL174
c1mim-mes-p1-mp2	-373.7803155	-42.58176379	-416.3620793	IL174
c1mim-ntf2-p1-mp2	-286.6024358	-63.94076488	-350.5432007	IL174
c1mim-ntf2-p2-mp2	-302.6269887	-35.66836056	-338.2953493	IL174
c1mim-ntf2-p3-mp2	-301.1592907	-41.42927435	-342.5885651	IL174
c1mim-pf6-p1-mp2	-320.2011643	-36.27730776	-356.4784721	IL174
c1mim-tos-p1-mp2	-363.6445524	-41.00495655	-404.6495089	IL174
c1mpyr-bf4-p1-mp2	-338.5081914	-32.73700622	-371.2451976	IL174
c1mpyr-bf4-p2-mp2	-332.8043758	-30.32967875	-363.1340545	IL174
c1mpyr-br-p1-mp2	-340.8835645	-43.52216654	-384.405731	IL174
c1mpyr-br-p2-mp2	-337.1455689	-41.84853766	-378.9941066	IL174
c1mpyr-cl-p1-mp2	-363.0846547	-37.77881609	-400.8634708	IL174
c1mpyr-cl-p2-mp2	-359.3909025	-36.97910415	-396.3700067	IL174
c1mpyr-dca-p1-mp2	-310.9721442	-39.69699754	-350.6691418	IL174
c1mpyr-dca-p2-mp2	-313.7640401	-43.21578964	-356.9798297	IL174
c1mpyr-dca-p3-mp2	-295.9708965	-39.67313144	-335.644028	IL174
c1mpyr-dca-p4-mp2	-314.234801	-41.81373462	-356.0485357	IL174
c1mpyr-mes-p1-mp2	-367.0218925	-38.32608329	-405.3479758	IL174
c1mpyr-mes-p2-mp2	-358.0771306	-34.06977254	-392.1469031	IL174
c1mpyr-ntf2-p1-mp2	-289.7462468	-43.54179243	-333.2880392	IL174
c1mpyr-ntf2-p2-mp2	-292.9118204	-34.67942323	-327.5912436	IL174
c1mpyr-ntf2-p3-mp2	-288.9257223	-42.40533035	-331.3310527	IL174
c1mpyr-ntf2-p5-mp2	-293.9613966	-36.00261195	-329.9640085	IL174
c1mpyr-pf6-p1-mp2	-314.16912	-32.32693992	-346.4960599	IL174
c1mpyr-pf6-p2-mp2	-309.5051794	-30.11317134	-339.6183507	IL174
c1mpyr-tos-p1-mp2	-359.222364	-36.43519628	-395.6575603	IL174
c1mpyr-tos-p2-mp2	-351.3668035	-32.12201707	-383.4888206	IL174
c2mim-bf4-p1-mp2	-342.8321619	-36.73997541	-379.5721373	IL174
c2mim-bf4-p2-mp2	-339.1398707	-34.23816261	-373.3780333	IL174

System	HF Energy	Correlation Energy	Total Interaction Energy	Suite
c2mim-br-p1-mp2	-345.7184724	-46.96958392	-392.6880563	IL174
c2mim-br-p2-mp2	-353.92933	-33.41679387	-387.3461238	IL174
c2mim-br-p3-mp2	-349.529642	-34.03038949	-383.5600315	IL174
c2mim-br-p4-mp2	-351.0398772	-46.47528589	-397.5151631	IL174
c2mim-cl-p1-mp2	-367.996253	-40.32376459	-408.3200176	IL174
c2mim-cl-p2-mp2	-378.7304438	-29.61895395	-408.3493977	IL174
c2mim-cl-p3-mp2	-373.4905473	-30.49692589	-403.9874732	IL174
c2mim-cl-p4-mp2	-372.2947895	-39.32103516	-411.6158246	IL174
c2mim-dca-p1-mp2	-308.1775648	-56.76969959	-364.9472644	IL174
c2mim-dca-p2-mp2	-309.4523866	-53.82443785	-363.2768245	IL174
c2mim-dca-p3-mp2	-310.2755572	-56.92446007	-367.2000173	IL174
c2mim-dca-p4-mp2	-309.70296	-54.24742256	-363.9503826	IL174
c2mim-dca-p5-mp2	-306.4727849	-55.69091435	-362.1636992	IL174
c2mim-dca-p6-mp2	-303.6419397	-55.79152719	-359.4334669	IL174
c2mim-mes-p1-mp2	-368.7574206	-47.44082118	-416.1982418	IL174
c2mim-mes-p2-mp2	-366.8349145	-43.14191336	-409.9768278	IL174
c2mim-ntf2-p1-mp2	-281.5427013	-70.03429014	-351.5769914	IL174
c2mim-ntf2-p2-mp2	-295.5996454	-45.32592844	-340.9255738	IL174
c2mim-ntf2-p3-mp2	-298.104376	-44.542232	-342.646608	IL174
c2mim-ntf2-p4-mp2	-281.633378	-61.74520469	-343.3785827	IL174
c2mim-pf6-p1-mp2	-316.7857779	-40.05361175	-356.8393897	IL174
c2mim-pf6-p2-mp2	-313.8757477	-36.45021504	-350.3259627	IL174
c2mim-tos-p1-mp2	-359.4476704	-46.90862183	-406.3562922	IL174
c2mim-tos-p2-mp2	-357.5787881	-41.82852089	-399.407309	IL174
c2mpyr-bf4-p1-mp2	-335.0198103	-33.19101064	-368.2108209	IL174
c2mpyr-bf4-p2-mp2	-322.6012955	-31.22033589	-353.8216313	IL174
c2mpyr-bf4-p3-mp2	-328.8587842	-30.93291425	-359.7916984	IL174
c2mpyr-br-p1-mp2	-336.5936497	-44.10413507	-380.6977848	IL174
c2mpyr-br-p2-mp2	-323.3780752	-42.82968237	-366.2077576	IL174
c2mpyr-br-p3-mp2	-331.9537613	-42.42933928	-374.3831005	IL174
c2mpyr-cl-p1-mp2	-358.3804895	-38.07097349	-396.4514629	IL174
c2mpyr-cl-p2-mp2	-344.6723388	-37.25865661	-381.9309954	IL174
c2mpyr-cl-p3-mp2	-354.0097088	-37.10314942	-391.1128583	IL174
c2mpyr-dca-p1-mp2	-306.1292976	-39.64286363	-345.7721612	IL174
c2mpyr-dca-p2-mp2	-306.4102009	-42.70233743	-349.1125384	IL174
c2mpyr-dca-p3-mp2	-306.0942419	-43.41796094	-349.5122028	IL174

System	HF Energy	Correlation Energy	Total Interaction Energy	Suite
c2mpyr-mes-p1-mp2	-363.9781104	-39.34294044	-403.3210508	IL174
c2mpyr-mes-p2-mp2	-350.9822872	-36.45470911	-387.4369963	IL174
c2mpyr-mes-p3-mp2	-355.5161142	-35.25750103	-390.7736152	IL174
c2mpyr-ntf2-p1-mp2	-287.4139613	-44.66575738	-332.0797187	IL174
c2mpyr-ntf2-p2-mp2	-287.8951862	-36.81403056	-324.7092167	IL174
c2mpyr-ntf2-p3-mp2	-280.6199463	-43.93645619	-324.5564025	IL174
c2mpyr-ntf2-p4-mp2	-284.169311	-42.93683332	-327.1061443	IL174
c2mpyr-ntf2-p5-mp2	-282.5553769	-38.41662734	-320.9720042	IL174
c2mpyr-ntf2-p6-mp2	-286.8566778	-36.31547581	-323.1721536	IL174
c2mpyr-pf6-p1-mp2	-308.6251647	-32.50600536	-341.1311701	IL174
c2mpyr-pf6-p2-mp2	-299.1202598	-30.93111833	-330.0513781	IL174
c2mpyr-pf6-p3-mp2	-305.5020039	-30.55192422	-336.0539282	IL174
c2mpyr-tos-p1-mp2	-355.9827609	-37.61719676	-393.5999577	IL174
c2mpyr-tos-p2-mp2	-343.0140547	-34.89014986	-377.9042046	IL174
c3mim-bf4-p1-mp2	-341.7767767	-37.49525975	-379.2720365	IL174
c3mim-bf4-p2-mp2	-340.9831918	-36.3451419	-377.3283337	IL174
c3mim-br-p1-mp2	-344.3393275	-47.5197504	-391.8590779	IL174
c3mim-br-p2-mp2	-351.7599327	-33.72882238	-385.4887551	IL174
c3mim-br-p3-mp2	-347.0420794	-37.81795428	-384.8600337	IL174
c3mim-br-p4-mp2	-351.1336111	-47.72426404	-398.8578752	IL174
c3mim-cl-p1-mp2	-365.7573438	-40.69998126	-406.457325	IL174
c3mim-cl-p2-mp2	-376.4840652	-29.73856445	-406.2226296	IL174
c3mim-cl-p3-mp2	-371.3049273	-33.26838878	-404.573316	IL174
c3mim-cl-p4-mp2	-372.316596	-40.10299595	-412.419592	IL174
c3mim-dca-p1-mp2	-305.2524173	-57.54243619	-362.7948535	IL174
c3mim-dca-p2-mp2	-302.5147562	-58.90073928	-361.4154955	IL174
c3mim-dca-p3-mp2	-306.5538507	-51.04233752	-357.5961882	IL174
c3mim-dca-p4-mp2	-309.8547533	-59.86400879	-369.7187621	IL174
c3mim-dca-p5-mp2	-303.5000037	-56.9995504	-360.4995541	IL174
c3mim-dca-p6-mp2	-300.6124202	-55.86982765	-356.4822478	IL174
c3mim-mes-p1-mp2	-368.1138884	-49.60972472	-417.7236132	IL174
c3mim-mes-p2-mp2	-363.7888757	-43.52658976	-407.3154655	IL174
c3mim-pf6-p1-mp2	-303.0144238	-40.40495079	-343.4193746	IL174
c3mim-pf6-p2-mp2	-311.1662901	-36.52358571	-347.6898758	IL174
c3mim-tos-p1-mp2	-358.2300826	-49.50737807	-407.7374606	IL174
c3mim-tos-p2-mp2	-356.7271662	-46.81377536	-403.5409416	IL174

System	HF Energy	Correlation Energy	Total Interaction Energy	Suite
c3mpyr-bf4-p1-mp2	-333.4846655	-33.52206047	-367.006726	IL174
c3mpyr-bf4-p2-mp2	-320.2104228	-31.37383379	-351.5842566	IL174
c3mpyr-bf4-p3-mp2	-327.0548462	-31.20056686	-358.2554131	IL174
c3mpyr-br-p1-mp2	-334.8651412	-44.81990349	-379.6850447	IL174
c3mpyr-br-p2-mp2	-320.1310655	-42.61336794	-362.7444334	IL174
c3mpyr-br-p3-mp2	-330.063278	-43.04477435	-373.1080523	IL174
c3mpyr-cl-p1-mp2	-356.6333211	-38.44944844	-395.0827695	IL174
c3mpyr-cl-p2-mp2	-341.9743804	-37.43897787	-379.4133582	IL174
c3mpyr-cl-p3-mp2	-351.9765684	-37.38002336	-389.3565918	IL174
c3mpyr-dca-p1-mp2	-305.9571047	-40.61336734	-346.570472	IL174
c3mpyr-dca-p2-mp2	-304.0193723	-42.67075368	-346.690126	IL174
c3mpyr-dca-p3-mp2	-308.2300699	-38.60306656	-346.8331364	IL174
c3mpyr-dca-p4-mp2	-283.8940486	-41.51279878	-325.4068474	IL174
c3mpyr-dca-p5-mp2	-303.1198608	-43.30314499	-346.4230058	IL174
c3mpyr-mes-p1-mp2	-362.5364678	-39.98966323	-402.5261311	IL174
c3mpyr-mes-p2-mp2	-348.2324219	-36.66117682	-384.8935987	IL174
c3mpyr-mes-p3-mp2	-353.8773315	-35.96540876	-389.8427403	IL174
c3mpyr-ntf2-p1-mp2	-284.7624852	-46.65771109	-331.4201963	IL174
c3mpyr-ntf2-p2-mp2	-283.5195815	-36.4220815	-319.941663	IL174
c3mpyr-ntf2-p3-mp2	-278.067281	-44.1139107	-322.1811917	IL174
c3mpyr-ntf2-p4-mp2	-282.7898685	-44.78694929	-327.5768177	IL174
c3mpyr-ntf2-p5-mp2	-280.9533434	-38.3265928	-319.2799362	IL174
c3mpyr-ntf2-p6-mp2	-284.4021024	-37.77012701	-322.1722294	IL174
c3mpyr-pf6-p1-mp2	-309.3692276	-33.57132413	-342.9405517	IL174
c3mpyr-pf6-p2-mp2	-296.8241521	-30.85357007	-327.6777221	IL174
c3mpyr-pf6-p3-mp2	-303.8627404	-30.99116145	-334.8539018	IL174
c3mpyr-tos-p1-mp2	-354.550237	-38.37123672	-392.9214737	IL174
c3mpyr-tos-p2-mp2	-340.2435155	-35.00884058	-375.2523561	IL174
c4mim-bf4-p1-mp2	-340.8415501	-37.95657694	-378.7981271	IL174
c4mim-bf4-p2-mp2	-339.6633872	-36.49994061	-376.1633279	IL174
c4mim-br-p1-mp2	-343.4712585	-48.25574338	-391.7270018	IL174
c4mim-br-p2-mp2	-351.0981555	-34.53499247	-385.633148	IL174
c4mim-br-p3-mp2	-345.5789449	-39.33749419	-384.9164391	IL174
c4mim-br-p4-mp2	-349.4956342	-48.11103553	-397.6066697	IL174
c4mim-cl-p1-mp2	-364.8455736	-41.15425441	-405.9998281	IL174
c4mim-cl-p2-mp2	-375.8364324	-30.1827531	-406.0191855	IL174

System	HF Energy	Correlation Energy	Total Interaction Energy	Suite
c4mim-cl-p3-mp2	-369.9503847	-34.39107577	-404.3414605	IL174
c4mim-cl-p4-mp2	-370.6844794	-40.41146036	-411.0959398	IL174
c4mim-dca-p1-mp2	-304.2078332	-58.07943587	-362.2872691	IL174
c4mim-dca-p2-mp2	-308.5205398	-61.15918168	-369.6797215	IL174
c4mim-dca-p3-mp2	-302.1138507	-57.22230351	-359.3361542	IL174
c4mim-mes-p1-mp2	-366.550917	-50.6537313	-417.2046483	IL174
c4mim-mes-p2-mp2	-362.0099881	-43.64231432	-405.6523024	IL174
c4mim-pf6-p1-mp2	-314.6492278	-42.57971105	-357.2289388	IL174
c4mim-pf6-p2-mp2	-309.7874123	-36.57442582	-346.3618382	IL174
c4mim-tos-p1-mp2	-356.5264758	-51.32876831	-407.8552442	IL174
c4mim-tos-p2-mp2	-357.1908505	-47.55323457	-404.744085	IL174
c4mpyr-bf4-p1-mp2	-332.0884959	-33.61903072	-365.7075266	IL174
c4mpyr-bf4-p2-mp2	-318.8174846	-31.47678409	-350.2942687	IL174
c4mpyr-bf4-p3-mp2	-325.680802	-31.40151882	-357.0823208	IL174
c4mpyr-br-p1-mp2	-333.3159505	-45.02527274	-378.3412232	IL174
c4mpyr-br-p2-mp2	-318.6915003	-42.81761782	-361.5091181	IL174
c4mpyr-br-p3-mp2	-328.43482	-43.27885784	-371.7136778	IL174
c4mpyr-cl-p1-mp2	-355.0206698	-38.45244524	-393.473115	IL174
c4mpyr-cl-p2-mp2	-340.9695577	-37.83424867	-378.8038064	IL174
c4mpyr-cl-p3-mp2	-350.4061407	-37.34728108	-387.7534217	IL174
c4mpyr-dca-p1-mp2	-301.283048	-40.41750046	-341.7005485	IL174
c4mpyr-dca-p2-mp2	-302.4945244	-42.61530321	-345.1098276	IL174
c4mpyr-dca-p3-mp2	-305.3687797	-41.27105576	-346.6398355	IL174
c4mpyr-dca-p4-mp2	-302.491139	-42.12580485	-344.6169439	IL174
c4mpyr-dca-p5-mp2	-304.5324114	-43.41471637	-347.9471278	IL174
c4mpyr-mes-p1-mp2	-360.8719445	-40.00125408	-400.8731986	IL174
c4mpyr-mes-p2-mp2	-346.7330966	-37.02600965	-383.7591063	IL174
c4mpyr-mes-p3-mp2	-352.3204774	-36.17194436	-388.4924218	IL174
c4mpyr-pf6-p1-mp2	-307.9381807	-33.66086331	-341.599044	IL174
c4mpyr-pf6-p2-mp2	-295.5351299	-31.23716226	-326.7722921	IL174
c4mpyr-pf6-p3-mp2	-302.752601	-31.20153379	-333.9541348	IL174
DMEA-CF3SO3-p1-opt	-375.3410607	-37.58794506	-412.9290058	HBIL
DMEA-Cl-p1-opt	-446.753835	-39.73330932	-486.4871443	HBIL
DMEA-mOSO3-p1-opt	-418.1590779	-36.95491686	-455.1139947	HBIL
DMEA-mSO3-p1-opt	-436.4993136	-38.61318743	-475.112501	HBIL
DMEA-NO3-p1-opt	-429.1176549	-43.11224092	-472.2298959	HBIL

System	HF Energy	Correlation Energy	Total Interaction Energy	Suite
DMEA-TFA-p1-opt	-442.8573378	-39.60465521	-482.461993	HBIL
EtMeNH2-mSO3-p1-opt	-455.9588635	-35.66597065	-491.6248341	HBIL
EtMeNH2-Cl-p1-opt	-454.6723876	-37.00770835	-491.680096	HBIL
EtMeNH2-mOSO3-p1-opt	-425.1357555	-48.6217696	-473.7575251	HBIL
EtMeNH2-NO3-p1-opt	-442.7530485	-38.83296889	-481.5860174	HBIL
EtMeNH2-TFA-p1-opt	-457.3278616	-35.02316088	-492.3510225	HBIL
EtMeNH2-CF3SO3-p1-opt	-397.8193081	-40.42444994	-438.243758	HBIL
EtNH3-CF3SO3-p1-opt	-414.4867784	-36.86031283	-451.3470912	HBIL
EtNH3-Cl-p1-opt	-468.8787423	-36.11558937	-504.9943317	HBIL
EtNH3-mOSO3-p1-opt	-438.7957633	-42.24869282	-481.0444561	HBIL
EtNH3-mSO3-p1-opt	-463.7931465	-37.30514263	-501.0982891	HBIL
EtNH3-NO3-p1-opt	-456.6924964	-40.18305154	-496.875548	HBIL
EtNH3-TFA-p1-opt	-482.7433835	-30.86555385	-513.6089373	HBIL
mim-CF3SO3-p1-opt	-329.837684	-31.11852008	-360.9562041	HBIL
mim-CF3SO3-p2-opt	-379.3150624	-27.24599236	-406.5610548	HBIL
mim-Cl-p1-opt	-415.9372312	-31.66378968	-447.6010208	HBIL
mim-Cl-p2-opt	-376.7058523	-20.21155857	-396.9174109	HBIL
mim-mOSO3-p1-opt	-340.2884479	-31.82095305	-372.1094009	HBIL
mim-mSO3-p1-opt	-434.4546613	-25.93040073	-460.3850621	HBIL
mim-mSO3-p2-opt	-378.9090002	-27.83513479	-406.744135	HBIL
mim-NO3-p1-opt	-424.8539015	-33.43629675	-458.2901982	HBIL
mim-NO3-p2-opt	-339.6096028	-44.71380379	-384.3234066	HBIL
mim-TFA-p1-opt	-371.5004746	-16.27260733	-387.7730819	HBIL
mim-TFA-p2-opt	-262.3273749	-17.93536303	-280.262738	HBIL
mim-TFA-p3-opt	-286.0120829	-28.47375783	-314.4858407	HBIL
mpyr-CF3SO3-p1-opt	-373.5773023	-46.58733837	-420.1646407	HBIL
mpyr-Cl-p1-opt	-444.7948032	-42.15602875	-486.950832	HBIL
mpyr-mOSO3-p1-opt	-404.2645724	-45.17533815	-449.4399106	HBIL
mpyr-mSO3-p1-opt	-432.3834049	-45.85062392	-478.2340288	HBIL
mpyr-NO3-p1-opt	-422.0118728	-47.08050925	-469.0923821	HBIL
mpyr-TFA-p1-opt	-404.6048806	-49.45840355	-454.0632841	HBIL
TMEA-CF3SO3-p1-opt	-315.3561879	-35.0522706	-350.4084585	HBIL
TMEA-Cl-p1-opt	-361.2511461	-30.83515532	-392.0863015	HBIL
TMEA-mOSO3-p1-opt	-338.3760074	-35.05398904	-373.4299965	HBIL
TMEA-mSO3-p1-opt	-359.4472334	-33.42555552	-392.8727889	HBIL
TMEA-NO3-p1-opt	-340.9405643	-31.08921877	-372.0297831	HBIL

System	HF Energy	Correlation Energy	Total Interaction Energy	Suite
TMEA-TFA-p1-opt	-325.7657362	-35.12857328	-360.8943095	HBIL
